# DNA damage induced PARP‐1 overactivation confers paclitaxel‐induced neuropathic pain by regulating mitochondrial oxidative metabolism

**DOI:** 10.1111/cns.70012

**Published:** 2024-08-30

**Authors:** Meng‐meng Ge, Jun‐jie Hu, Ya‐qun Zhou, Yu‐ke Tian, Zhi‐heng Liu, Hui Yang, Yi‐rong Zhou, Qiu Qiu, Da‐wei Ye

**Affiliations:** ^1^ Department of Anesthesiology, Zhongshan Hospital Fudan University Shanghai China; ^2^ Department of Anesthesiology and Pain Medicine, Tongji Hospital Tongji Medical College, Huazhong University of Science and Technology Wuhan China; ^3^ Cancer Center, Tongji Hospital Tongji Medical College, Huazhong University of Science and Technology Wuhan China; ^4^ Department of Anesthesiology Shenzhen Second People's Hospital/The First Affiliated Hospital of Shenzhen University, Health Science Center Shenzhen China; ^5^ School of Pharmacy Tongji Medical College, Huazhong University of Science and Technology Wuhan China; ^6^ Department of Anaesthesiology Queen Mary Hospital, Hong Kong, Special Administrative Region Hong Kong China

**Keywords:** Olaparib PROTAC, oxidative metabolism, paclitaxel‐induced neuropathic pain, PARP‐1, Sirt‐3

## Abstract

**Aims:**

Poly (ADP‐ribose) polymerase (PARP) has been extensively investigated in human cancers. Recent studies verified that current available PARP inhibitors (Olaparib or Veliparib) provided clinical palliation of clinical patients suffering from paclitaxel‐induced neuropathic pain (PINP). However, the underlying mechanism of PARP overactivation in the development of PINP remains to be investigated.

**Methods and Results:**

We reported induction of DNA oxidative damage, PARP‐1 overactivation, and subsequent nicotinamide adenine dinucleotide (NAD^+^) depletion as crucial events in the pathogenesis of PINP. Therefore, we developed an Olaparib PROTAC to achieve the efficient degradation of PARP. Continuous intrathecal injection of Olaparib PROTAC protected against PINP by inhibiting the activity of PARP‐1 in rats. PARP‐1, but not PARP‐2, was shown to be a crucial enzyme in the development of PINP. Specific inhibition of PARP‐1 enhanced mitochondrial redox metabolism partly by upregulating the expression and deacetylase activity of sirtuin‐3 (SIRT3) in the dorsal root ganglions and spinal cord in the PINP rats. Moreover, an increase in the NAD^+^ level was found to be a crucial mechanism by which PARP‐1 inhibition enhanced SIRT3 activity.

**Conclusion:**

The findings provide a novel insight into the mechanism of DNA oxidative damage in the development of PINP and implicate PARP‐1 as a possible therapeutic target for clinical PINP treatment.

## Graphical abstract

Induction of DNA oxidative damage, PARP‐1 overactivation, and subsequent NAD^+^ depletion were proved to be crucial events in the pathogenesis of PINP. Specific inhibition of PARP‐1 enhanced mitochondrial redox metabolism partly by upregulating the expression and deacetylase activity of SIRT3 in the PINP rats.

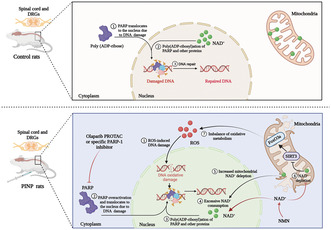

## 1 INTRODUCTION

Paclitaxel‐induced neuropathic pain (PINP) is a highly debilitating, dose‐limiting side effect of paclitaxel, and it affects the quality of life of cancer patients.^1^, ^2^ This painful neuropathy, which is characterized by paresthesia, dysesthesia, hyperalgesia, and allodynia, affects over 60% of patients in the first 3 months after paclitaxel treatment, and the drugs that used to prevent and treat PINP are not yet optimal.^3^, ^4^ Hence, it is critical to explore alternative therapeutic targets for the treatment of PINP.

Oxidative stress, characterized by excessive accumulation of reactive oxygen species (ROS) or decreased antioxidant system ability, plays a pivotal role in chronic pain pathophysiology.^5^, ^6^, ^7^ Mitochondria, as a major generator of cellular ROS, has also been demonstrated to be the primary targets of oxidative stress.^8^ ROS accumulation suppresses mitochondrial efficiency and results in the generation of more ROS in a vicious self‐destructive cycle, which in turn irreversibly results in lipid oxidation, protein disorder, and DNA damage.^9^, ^10^ Poly (ADP‐ribose) polymerase (PARP), as the primary enzyme for DNA damage response, has been extensively investigated in human cancers.^11^, ^12^ PARP‐1, as the most studied member of this protein family, undertakes the majority of PARylation (85–90%).^13^, ^14^ In addition to its crucial role as an oncology target, abnormal increase in the activity of PARP, especially PARP‐1, has been reported in conditions of neurodegenerative diseases, cancer, cardiovascular diseases, viral infections, and so on in preclinical studies.^15^, ^16^ Moreover, intrathecal administration of PARP1/2 inhibitor PJ‐34 or a specific PARP‐1 inhibitor TIQ‐A has represented protective effect on oxidative stress‐related disorders.^17^, ^18^ Recently, some research further verified that current available PARP inhibitors (Olaparib or Veliparib) provided clinical palliation of clinical patients suffering from PINP.^19^ However, the underlying mechanism of PARP overactivation in the development of PINP remains to be investigated.

On the basis of these previous observations, we explored the origin and consequences of PARP overactivation in rat model of PINP by characterizing the pathways involved and investigated the analgesic effects of PARP inhibitors. These findings reported that paclitaxel injection induced DNA oxidative damage, consequently resulting in PARP‐1 overactivation and nicotinamide adenine dinucleotide (NAD^+^) depletion. Specific inhibition of PARP‐1 enhanced mitochondrial redox metabolism partly by upregulating the deacetylase activity of sirtuin‐3 (SIRT3) in the dorsal root ganglions (DRGs) and spinal cord in the PINP rats. Moreover, an increase in the NAD^+^ levels was found to be a crucial mechanism by which PARP‐1 inhibition enhanced SIRT3 activity. Our study provides a novel insight into the mechanism of DNA oxidative damage in the development of PINP and implicates PARP‐1 as a possible therapeutic target for clinical PINP treatment.

## 2 MATERIALS AND METHODS

### 2.1 Animals

Male Sprague–Dawley rats (weighing 200–220 g) were obtained from Tongji Medical College, Huazhong University of Science and Technology, Wuhan, China. The rats were housed two per cage in a specific‐pathogen free environment (temperature: 22 ± 1°C, relative humidity: 40%–50%, 12 h light‐dark cycle). The experiments were carried out according to the Guide for the Use of Laboratory Animals and the National Institutes of Health Guide for the Care and approved by the Committee of Huazhong University of Science & Technology (approval number: TJH‐202103004).

### 2.2 Chemical synthesis of Olaparib PROTAC

The specific process of Olaparib PROTAC synthesis is shown in supplemental method 1—Data S1.

### 2.3 Reagents or resources

All the reagents or resources used in this study are presented in Table S1.

### 2.4 Paclitaxel‐induced neuropathic pain model

Paclitaxel solution (20 mg/mL diluted in dimethyl sulfoxide (DMSO)) was mixed with 5% Tween 80, 40% polyethylene glycol 300 (PEG300), and 45% ultrapure water to achieve a final concentration of 2 mg/ml.^20^ Paclitaxel (2 mg/kg) was intraperitoneally (i.p.) administered to the rats on 4 alternating days (days 0, 2, 4, and 6, cumulative dose of 8 mg/kg) as previously reported.^1^, ^21^

### 2.5 Behavioral tests

Mechanical allodynia was assessed by an individual who was blinded to the drug treatment during the mild‐light hours (10 a.m.–5 p.m.). Bilateral hind paw withdrawal thresholds (PWTs) in response to von Frey filament stimuli were evaluated with an up‐down method.^22^, ^23^ Filaments (1.4, 2, 4, 6, 8, 10, and 15 g) were ascendingly applied to the mid‐plantar of both hind paws for 6–8 s or until a paw was withdrawn from the filament. Positive responses were defined as a rapid withdrawal or licking of the hind paw after stimulation.

### 2.6 Intrathecal catheter implantation and drug administration

Under pentobarbital sodium (50 mg/kg, i.p.) and 2.5% isoflurane anesthesia, a polyethylene‐10 catheter (PE‐10, Anilab Software & Instruments, Ningbo, China) was inserted into the subarachnoid cavity in the L4‐L5 intervertebral space 5 days before PINP model establishment as previously described.^24^, ^25^, ^26^ Catheterization was considered to be successful when we observed temporary paralysis after the intrathecal administration of 2% lidocaine (10 μL). For drug preparation, Olaparib PROTAC (1, 5, or 10 μg/10 μL) and SIRT3 inhibitor 3‐TYP (150 μg/15 μL) were dissolved in ultrapure water supplemented with 10% DMSO and 20% PEG300. The specific PARP‐1 inhibitor AG14361 (5, 10, or 25 μg/10 μL) was dissolved in ultrapure water supplemented with 10% DMSO. The NAD^+^ precursor NMN (200 μg/10 μL or 750 μg/15 μL) was dissolved in normal saline. The specific drug injection schedules are shown in Figure S1.

### 2.7 Body weight and histopathology assessments

The body weight of each rat was recorded on days 0, 3, 7, 10, and 14 after paclitaxel injection using the same scale (0–2200 g, PTY‐B2200, Huazhi, USA). For histological analysis, tissue sections (45 μm) of rats' livers, kidneys, spleens, lungs, and hearts‐ were prepared and stained with hematoxylin and eosin (H&E). Then, the slides were examined and photographed with a light microscope (BX‐53, Olympus Optical, Tokyo, Japan). All the histological evaluations were made under blinded conditions.

### 2.8 Motor function assessment

#### 2.8.1 Open field test

The open field tests (OFTs) were performed based on previous studies.^27^, ^28^ The apparatus used for the OFTs is a square black plastic arena (60 cm × 60 cm × 40 cm). The rats were individually placed in the center of this arena. Each rat was subjected to the test only once, and the test lasted for 5 min. The total distance traveled and the average velocity of each rat were quantified by a video recording system (Labmaze V3.0, Zhongshidichuang Science and Technology Development Co., Ltd., Beijing, China).

### 2.9 Gait assessment

Rat gait during spontaneous ambulation was scored on a scale of I‐IV based on previous studies.^29^, ^30^ The specific graded standard is as follows: I = normal gait, II = abnormal gait, dysfunction of one hind limb, III = abnormal gait, dysfunction of both hind limbs but still able to walk, IV = paralysis or paraplegia.

### 2.10 Electron microscopy

Spinal cord tissues (1 mm^3^) were fixed in 2.5% glutaraldehyde at 4°C overnight. After being processed with a sequence of chemical treatments (1% osmium tetroxide, distilled water, etc.), the tissues were imbedded in epoxy resin monomer as previously described.^10^ Ultrathin sections were prepared and then stained with 2% uranyl acetate and lead citrate. The ultrastructure of the mitochondria in the spinal cord was scanned and photographed by a transmission electron microscopy (TEM) system (FEI Tecnai G20 TWIN, USA).

### 2.11 ROS, malondialdehyde (MDA), adenosine triphosphate (ATP), and NAD ^+^ content determination

The ROS, MDA, ATP, and NAD^+^ contents were measured by the ROS assay kit (Nanjing Jiancheng Bio‐Technology, Cat #E004‐1‐1), MDA assay kit (Nanjing Jiancheng Bio‐Technology, Cat #A003‐1), ATP assay kit (Nanjing Jiancheng Bio‐Technology, Cat #A095), and NAD^+^/NADH assay kit (Beyotime Biotechnology, Cat #S0175) based on the manufacturer's instructions, respectively.^31^, ^32^, ^33^, ^34^

### 2.12 Western blotting

L4‐L6 DRGs and spinal cord samples were lysed in cold radioimmunoprecipitation assay buffer supplemented with proteinase and phosphatase inhibitors (Boster, Wuhan, China). The protein concentration was determined by the bicinchoninic acid (BCA) protein assay kit (Boster, Cat #AR0146). Equal amounts of protein (40 μg) were separated by SDS‐PAGE, transferred to polyvinylidene fluoride membranes (Millipore, Billerica, MA, USA), and incubated with primary antibodies targeting PARP‐1 (1:1000, Cat #A0942, ABclonal; 1:1000, Cat #9532, Cell Signaling Technology), PARP‐2 (1:500, Cat #A16475, ABclonal), p‐γH2A.X (Ser 139) (1:500, Cat #9718, Cell Signaling Technology), SIRT3 (1:1000, Cat #AF5135, Affinity Biosciences; 1:1000 Cat #2627, Cell Signaling Technology), manganese superoxide dismutase (SOD2) (1:2000, Cat #A1340, ABclonal), Catalase (1:1000, Cat #A11777, ABclonal), Ac‐SOD2 (1:1000, Cat #AF3751, Affinity Biosciences), p‐FoxO3a (1:1000, Cat #AP0684, Abclonal; 1:1000, Cat #AF3020, Affinity Biosciences), and FoxO3a (1:1000, Cat #A0120, ABclonal). β‐actin (1:2000, Cat #AC026, ABclonal) was used to quantify the intensity of the protein bands, and protein expression is expressed as the fold change relative to the control.

### 2.13 Immunohistochemistry

Cryostat sections of transverse DRG and spinal cord samples were permeabilized with 0.3% Triton X‐100 and then incubated with 10% goat serum (except for ionized calcium‐binding adaptor molecule‐1 (Iba‐1)‐treated spinal cord sections, which was blocked with 10% donkey serum) at room temperature. For immunofluorescence staining, the slides were incubated with the following primary antibodies: mouse anti‐p‐γH2A.X (Ser 139) (1:50, Cat #ab26350, Abcam) and mouse anti‐8‐hydroxy‐2'‐deoxyguanosine (8‐OHdG) (1:100, Cat #ab48508, Abcam). Then, the slides were incubated with an Alexa Fluor 488‐conjugated goat antimouse secondary antibody. For double immunofluorescence staining, the slides were incubated with two primary antibodies (PARP‐1/SIRT3 + neurofilament‐200 (NF‐200), PARP‐1/SIRT3 + calcitonin gene‐related peptide (CGRP), PARP‐1/SIRT3 + IB4, PARP‐1/SIRT3 + glial fibrillary acidic protein (GFAP)), followed by two corresponding secondary antibodies. Then, the slides were stained with 4,6‐diamidino‐2‐phenylindole and imaged with an Olympus BX51 fluorescence microscope (Olympus Optical, Tokyo, Japan). The mean fluorescence intensities of p‐γH2A.X (Ser 139) and 8‐OHdG were analyzed using ImageJ as previously described.^35^

### 2.14 Bioinformatics analysis

A protein–protein interaction (PPI) network was constructed with the STRING database, and the interactions among PARP‐1, SIRT3, and p‐FOXO3a were analyzed with Cytoscape software. The Kyoto Encyclopedia of Genes and Genomes (KEGG) database was used to analyze the related signaling pathways.

### 2.15 Statistics

Statistical analysis was performed using GraphPad Prism 8.0.2 (GraphPad Software, San Diego, CA, USA). All the data was expressed as the mean ± SEM. Data normality was analyzed with Shapiro–Wilk test. *P*>0.05 was considered to indicate a normal distribution. Otherwise, nonparametric tests were used. Kruskal‐Wallis test was performed for multiple group statistical evaluation followed by Dunn's multiple comparisons test. If data conformed to the sample variance homogeneity assumption, statistical significance was performed by unpaired Student's *t* test for two groups; For multiple groups, one‐way analysis of variance with repeated measures followed by the Bonferroni post hoc test was used. The behavioral data among groups were analyzed by two‐way analysis of variance with repeated measures followed by Bonferroni post hoc tests. *p* < 0.05 was considered to indicate statistical significance.

## 3 RESULTS

### 3.1 Intraperitoneal administration of paclitaxel resulted in mechanical allodynia as well as DNA oxidative damage in rats

**FIGURE 1:**
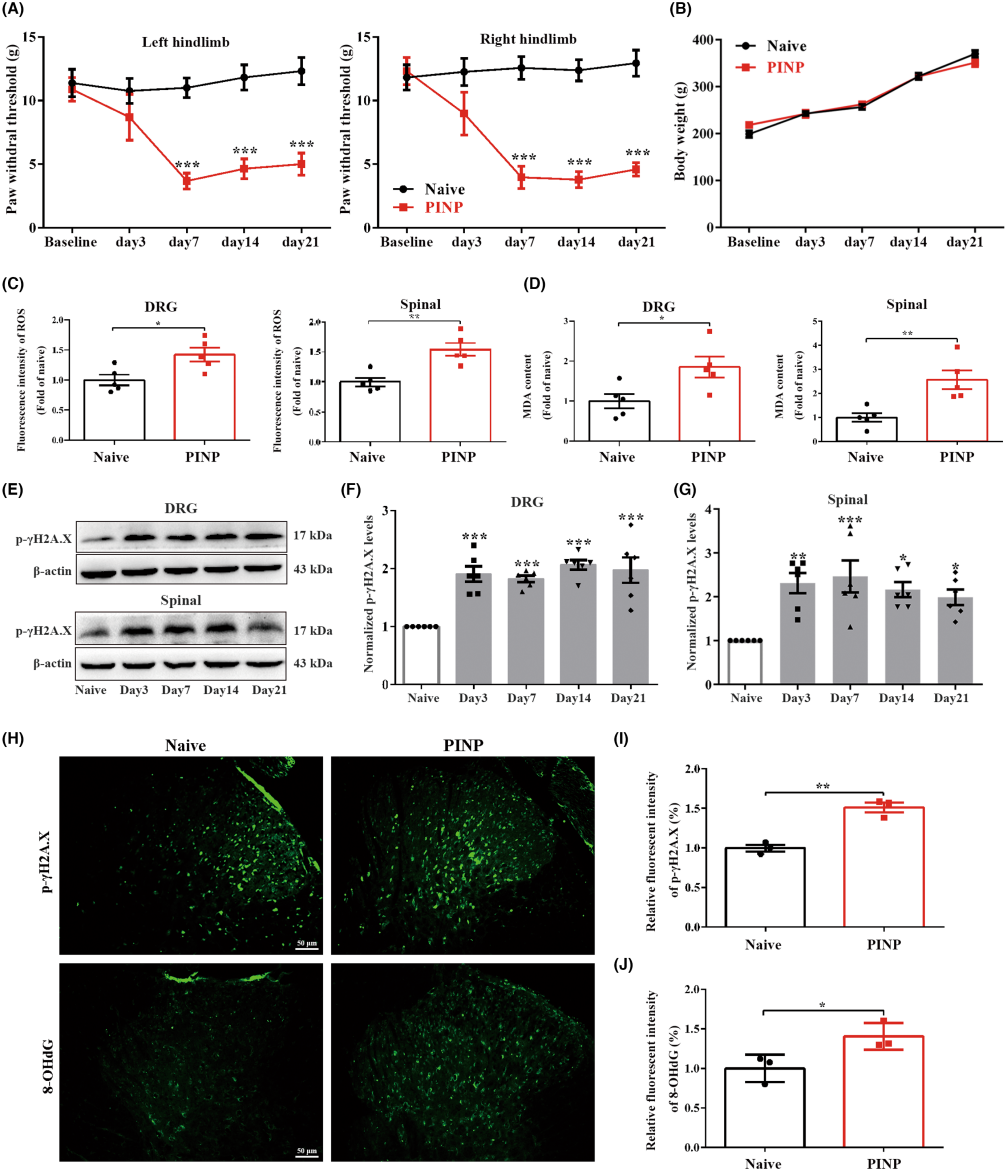
Intraperitoneal administration of paclitaxel results in mechanical allodynia as well as DNA oxidative damage in rats. (A) Paclitaxel injection induced a remarkable reduction in PWTs on the bilateral hindlimbs. Two‐way ANOVA followed by Bonferroni post hoc test, ***p < 0.001 versus Naïve group, *n* = 8 per group. (B) The body weight showed no remarkable differences in the Naïve and PINP groups. Two‐way ANOVA followed by Bonferroni post hoc test, *p* > 0.05, *n* = 8 per group. (C, D) Paclitaxel injection resulted in the upregulation of ROS and MDA levels in the DRGs and spinal cord. Normalized to Naïve group. Unpaired Student's *t*‐test, *p < 0.05, **p < 0.01 versus Naïve group, *n* = 5 per group. (E–G) The levels of p‐γH2A.X (Ser 139) were increased in the DRGs and spinal cord in the PINP group. One‐way ANOVA followed by Bonferroni post hoc test, *p < 0.05, **p < 0.01, ***p < 0.001 versus Naïve group, *n* = 6 per group. (H–J) Relative immunofluorescence staining intensities of p‐γH2A.X (Ser 139) and 8‐OHdG in the spinal cord dorsal horn in each group. Normalized to Naïve group. Unpaired Student's *t*‐test, *p < 0.05, **p < 0.01 versus Naïve group, *n* = 3 per group. Scale bar: 50 μm.

In line with our previous study,^1^, ^20^ the intraperitoneal administration of paclitaxel (2 mg/kg/day, 4 alternate days) markedly decreased the PWTs of the bilateral hind paws from day 7 to the last observation day (day 21) (Figure 1A). No significant differences in body weight were observed between Naïve and PINP groups (Figure 1B). All the data suggested that paclitaxel injection induced a stable mechanical allodynia, but did not influence the condition of rats.

Our previous study demonstrated that excessive ROS generation is crucial for the development of cancer‐induced bone pain.^6^ Consistently, we also observed that paclitaxel injection markedly upregulated the levels of ROS and MDA (a final product of lipid peroxidation) in the DRGs and spinal cord of rats (Figure 1C,D). Excessive ROS accumulation has been previously shown to contribute to DNA oxidative damage.^36^, ^37^ To further verify the effect of paclitaxel injection on DNA oxidative damage in rats, the expression of p‐γH2A.X (Ser 139), which is a sensitive biomarker for DNA double‐strand breaks, was analyzed. As expected, the protein expressions of p‐γH2A.X (Ser 139) were increased in the DRGs and spinal cord in the PINP rats (Figure 1E–G). Moreover, the immunofluorescence staining analysis of p‐γH2A.X (Ser 139) and 8‐OHdG (one of the major forms of free radical‐induced oxidative lesions) was markedly increased in the spinal cord dorsal horn in the PINP group, providing additional evidence that supported our western blot result (Figure 1H–J).

### 3.2 PARP‐1 is a crucial enzyme participating in the development of PINP in rats

**FIGURE 2:**
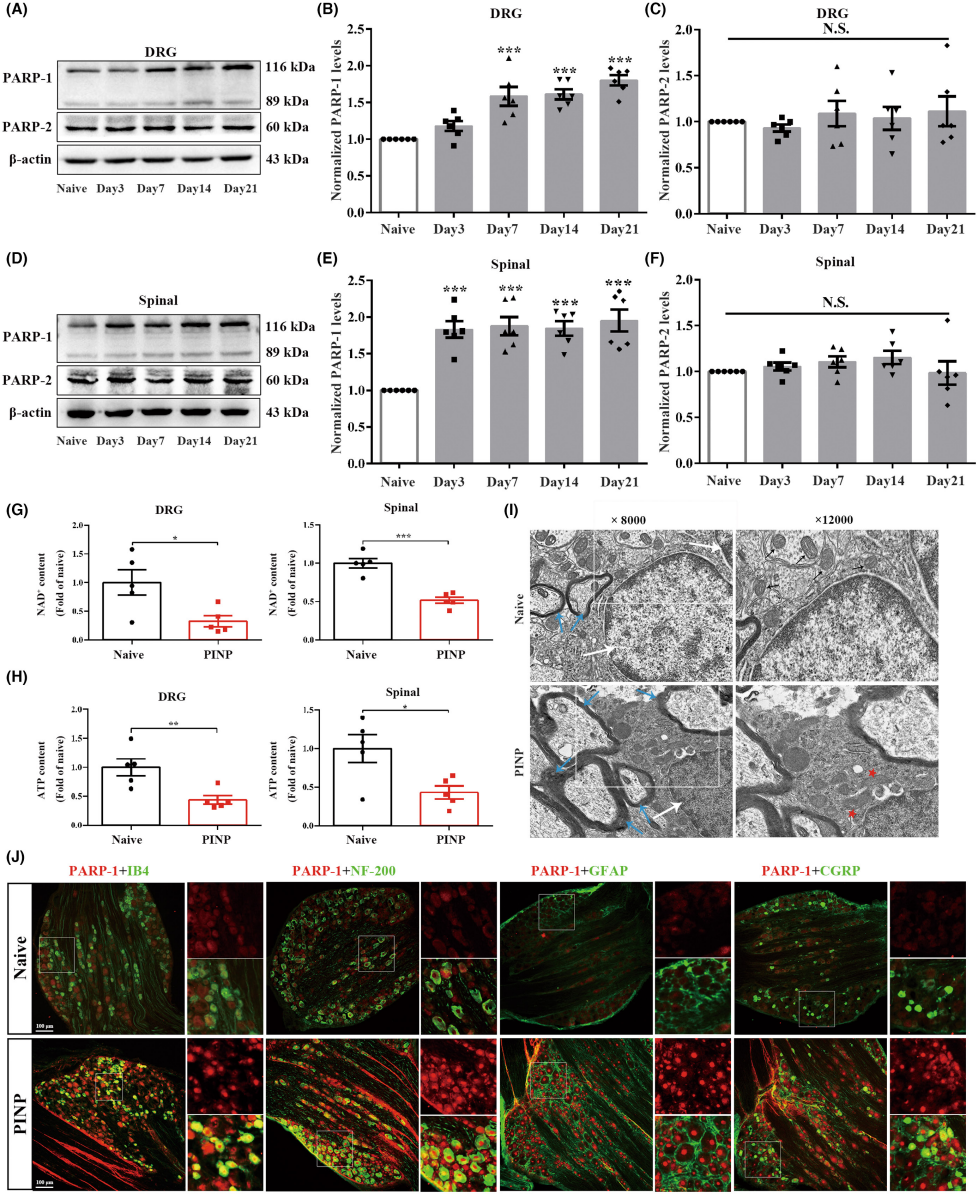
Intraperitoneal administration of paclitaxel induced PARP‐1 upregulations in DRGs and spinal cord of rats. (A–F) Paclitaxel injection‐induced a remarkable increased levels of PARP‐1 in the DRGs and spinal cord. One‐way ANOVA followed by Bonferroni post hoc test, ***p < 0.001 versus Naïve group, *n* = 6 per group. While, we did not detect remarkable changes of protein levels of PARP‐2 in the DRGs and spinal cord in each group. One‐way ANOVA followed by Bonferroni post hoc test, *p* > 0.05, *n* = 6 per group. (G, H) NAD^+^ and ATP levels in the DRGs and spinal cord were decreased in the PINP rats. Normalized to Naïve group. Unpaired Student's *t*‐test, *p < 0.05, **p < 0.01, ***p < 0.001 versus Naïve group, *n* = 5 per group. (I) TEM images of mitochondrial ultrastructure (white arrow: Cell nucleus; blue arrow: Myelin sheath; black arrow: Normal mitochondria; red pentacle: Damaged mitochondria). The magnifications were 8000 or 12,000 times. (J) Representative double‐immunofluorescence staining of PARP‐1 and IB4, NF‐200, CGRP or GFAP in the DRGs in each group. Scale bar: 100 μm, *n* = 3 per group.

Previous studies have demonstrated that excessive accumulation of ROS could induce DNA oxidative damage, thus resulting in PARP overactivation in various disorders.^38^ We next analyzed the changes in the protein expression of these two major PARP subtypes in the DRGs and spinal cord of rats. The levels of PARP‐1 were markedly upregulated in the DRGs in the PINP rats (Figure 2A–C). However, no remarkable differences were observed in the PARP‐2 levels in each group (Figure 2A–C). The changes in the levels of PARP‐1 and PARP‐2 in the spinal cord showed trends that were similar to those in the DRGs in each group (Figure 2D–F). Severe stress‐induced PARP overactivation can lead to the depletion of NAD^+^, which subsequently results in the depletion of ATP.^16^, ^39^ As expected, the levels of NAD^+^ and ATP in the DRGs and spinal cord in the PINP group were decreased compared to those in the Naïve group (Figure 2G,H). Mitochondria, which are a crucial organelle for intracellular ATP production, are the primary target of oxidative stress.^40^ TEM showed significant changes in mitochondrial morphology in the spinal cord in the PINP group. As shown in Figure 2I, (1) the mitochondria that are labeled with asterisks exhibited seriously damaged mitochondrial crests and fractured mitochondrial membranes. (2) Shrinkage of the nuclear membrane and turbulence of the myelin sheath were observed in the PINP group. Furthermore, double immunofluorescence staining revealed that PARP‐1 was mostly colocalized with IB4‐labeled nonpeptidergic neurons, NF‐200‐labeled neurons, and CGRP‐labeled peptidergic neurons, but not with GFAP‐labeled satellite glial cells in the DRGs of rats in Naïve and PINP groups (Figure 2J).

### 3.3 Intrathecal injection of Olaparib PROTAC attenuated paclitaxel injection‐induced mechanical allodynia by inhibition of PARP‐1in rats

**FIGURE 3:**
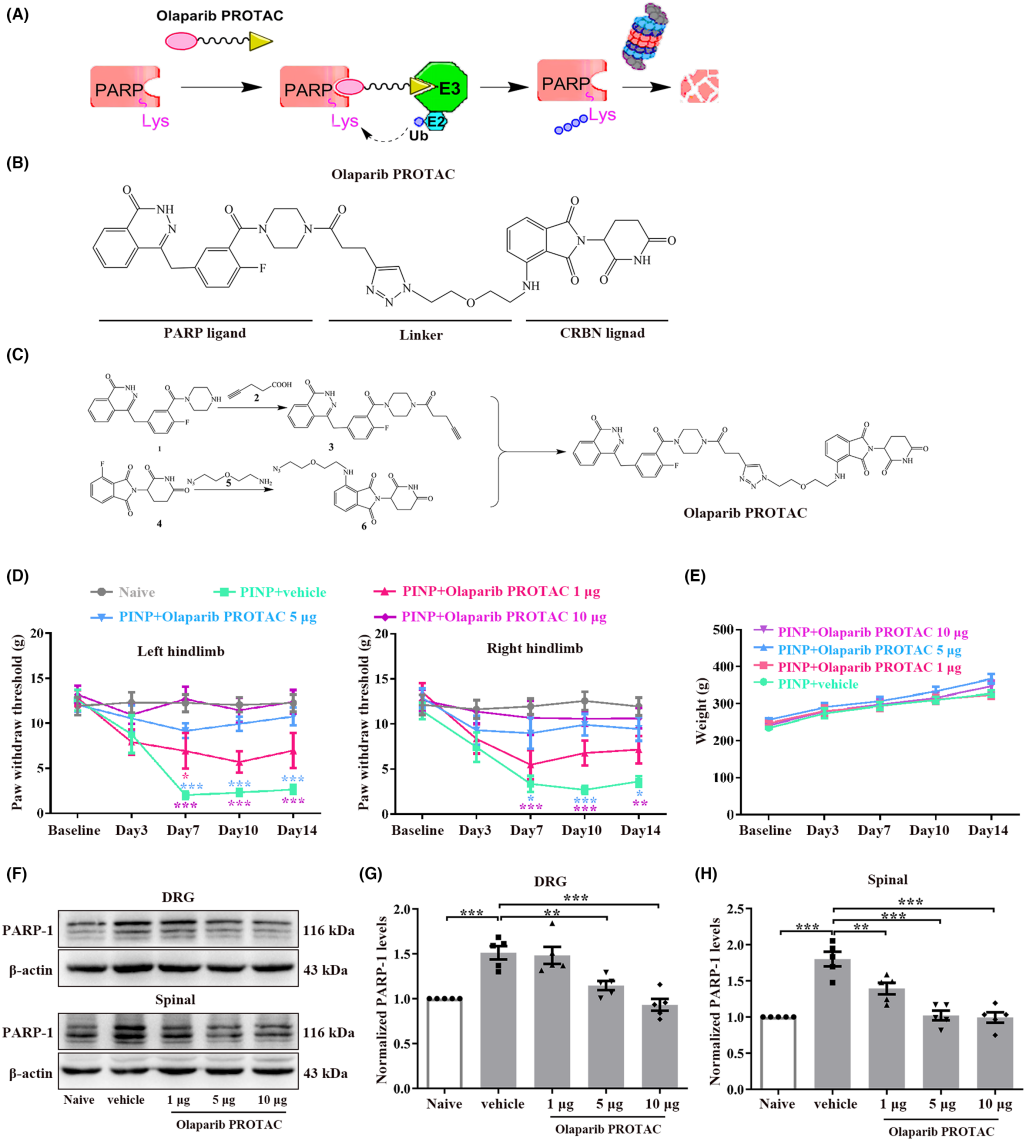
Continuous intrathecal injection of Olaparib PROTAC alleviated paclitaxel injection‐induced mechanical allodynia by inhibition of PARP‐1 in rats. (A) Working model of Olaparib PROTAC. (B) The designed Olaparib PROTAC composed of a PARP ligand, a linker and a CRBN ligand. (C) The details of Olaparib PROTAC synthesis. (D) Repeated intrathecal injection of Olaparib PROTAC (1, 5, or 10 μg/10 μL) reversed the decreased PWTs in a dose‐dependent manner. Two‐way ANOVA followed by Bonferroni post hoc test, *p < 0.05, **p < 0.01, ***p < 0.001 versus PINP+vehicle group, *n* = 7–8 per group. (E) No significant differences of the body weight were observed in PINP+Olaparib PROTAC groups compared to PINP+vehicle group. Two‐way ANOVA followed by Bonferroni post hoc test, *p* > 0.05, *n* = 7 per group. (F–H) Continuous intrathecal injection of Olaparib PROTAC inhibited the upregulations of PARP‐1 protein in the PINP rats in a dose‐dependent manner. One‐way ANOVA followed by Bonferroni post hoc test, **p < 0.01, ***p < 0.001 versus corresponding groups, *n* = 5 per group.

To achieve the selective and efficient proteolysis of PARP, we designed Olaparib PROTAC by coupling PARP with the CRBE ligand (Figure 3A–C). Next, we investigated whether intrathecal administration of Olaparib PROTAC alleviated PINP. Repeated intrathecal injection of Olaparib PROTAC (1, 5, or 10 μg/10 μL) once daily for 14 consecutive days reversed the decreased PWTs in a dose‐dependent manner (Figure 3D). Moreover, no significant differences in body weight were observed in the PINP+Olaparib PROTAC groups compared to the PINP+vehicle group, suggesting that Olaparib PROTAC did not affect the condition of rats (Figure 3E). We next explored whether Olaparib PROTAC exerted its analgesic effect by inhibiting the activation of PARP‐1 in the PINP rats. Continuous intrathecal injection of Olaparib PROTAC led to effective degradation of PARP‐1 in the DRGs and spinal cord in the PINP rats (Figure 3F–H). All these results suggested that Olaparib PROTAC inhibited the activation of PARP‐1 in the PINP rats.

**FIGURE 4:**
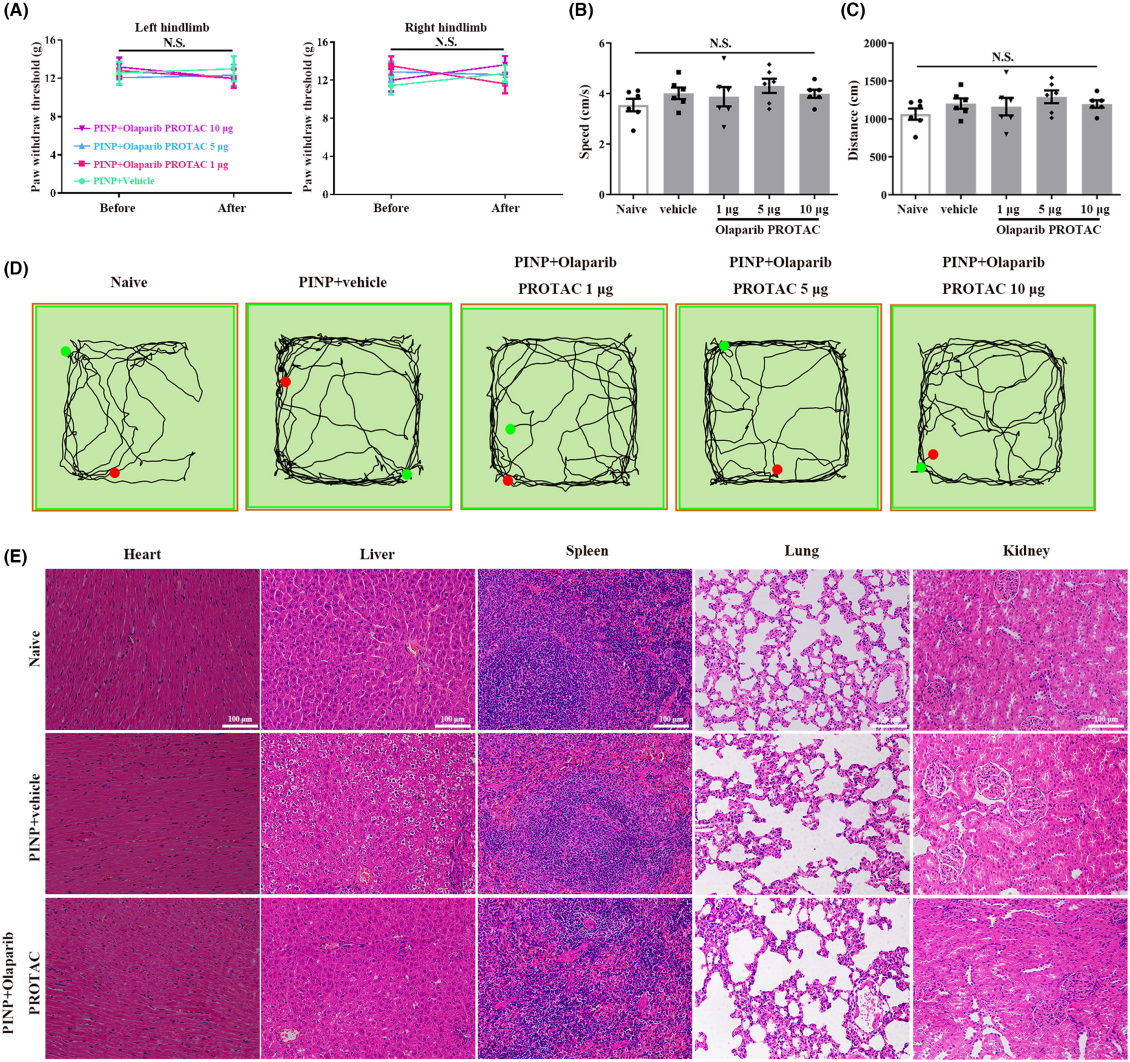
The effects of Olaparib PROTAC administration on motor function and organ structure for toxicity evaluation. (A) The PWTs before and after intrathecal catheter implantation revealed no remarkable differences on both hindlimbs in each group. Two‐way ANOVA followed by Bonferroni post hoc test, *p* > 0.05, *n* = 7–8 per group. (B–D) No remarkable differences in the total distance and average velocity were observed among all groups. One‐way ANOVA followed by Bonferroni post hoc test, *p* > 0.05, *n* = 6 per group. (E) Representative photomicrographs of H&E staining of heart, liver, spleen, lungs and kidneys. Scale bar: 100 μm, *n* = 3 per group.

We also explored the effect of Olaparib PROTAC on motor function, as well as the effects of intrathecal catheter implantation on pain behavior and motor function. The PWTs showed no significant differences in both hindlimbs when assessed before and after intrathecal catheter implantation in each group (Figure 4A). No remarkable differences were observed in the total distance and average velocity among the five groups (Figure 4B–D). In addition, the gait assessment results are as follows: I: 86.7% and II: 13.3% in the PINP+vehicle group; I: 80%, II: 13.3%, and III: 6.7% in the PINP+Olaparib PROTAC group (Table S2). All these results suggested that intrathecal catheter implantation and Olaparib PROTAC administration had no significant effects on motor function. Moreover, the toxic effects of Olaparib PROTAC were analyzed by histological examination. Representative images of solid organs are shown in Figure 4E. No abnormalities were observed in the heart, spleen, or lung in any group. For the liver, no significant hydropic degeneration was observed in each group. In addition, the structures of the renal cortex, medulla, and corpuscles were normal (Figure 4E).

### 3.4 Intrathecal injection of a specific PARP‐1 inhibitor attenuated paclitaxel injection‐induced mechanical allodynia in rats

**FIGURE 5:**
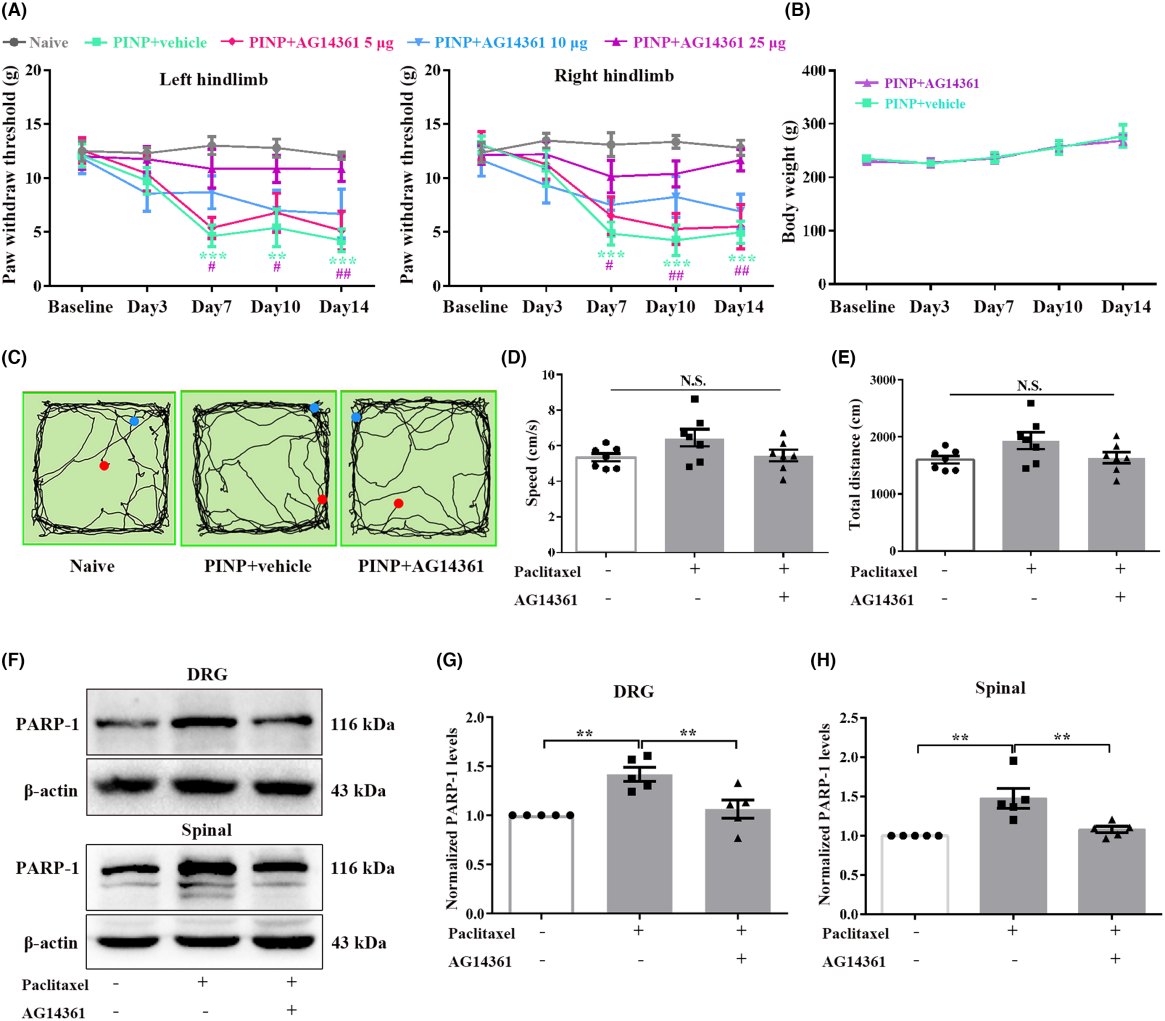
Continuous intrathecal injection of a specific PARP‐1 inhibitor alleviated PINP in rats. (A) The decreased PWTs in the PINP group were partially reversed by injection of AG14361 (25 μg/10 μL). Two‐way ANOVA followed by Bonferroni post hoc test, **p < 0.01, ***p < 0.001 PINP+Vehicle group versus Naïve group, #*p* < 0.05, ##*p* < 0.01 PINP+AG14361 25 μg group versus PINP+vehicle group, *n* = 6–7 per group. (B) No significant differences of the body weight were observed in PINP+AG14361 groups compared to PINP+vehicle group. Two‐way ANOVA followed by Bonferroni post hoc test, *p* > 0.05, *n* = 7 per group. (C–E) There were no remarkable differences in the total distance and average velocity among three groups. One‐way ANOVA followed by Bonferroni post hoc test, *p* > 0.05, *n* = 7 per group. (F–H) Continuous intrathecal administration of AG14361 (25 μg/10 μL) inhibited the upregulations of PARP‐1 proteins in the DRGs and spinal cord in the PINP rats. one‐way ANOVA followed by Bonferroni post hoc test, **p < 0.01 versus corresponding groups, *n* = 5 per group.

To further determine the potential role of PARP‐1 in the development of PINP, the action of a specific PARP‐1 inhibitor AG14361 was examined. Repeated intrathecal administration of AG14361 (5, 10, or 25 μg/10 μL) once daily for 14 consecutive days alleviated mechanical allodynia in a dose‐dependent manner. The decreased PWTs in the PINP group were partially reversed by injection of AG14361 (25 μg/10 μL) (Figure 5A). There were no notable differences in body weight in the PINP+AG14361 group compared to the PINP+vehicle group (Figure 5B). In addition, the OFT results revealed that intrathecal catheter implantation and AG14361 administration had no effects on motor function of rats (Figure 5C–E). Western blot results demonstrated that repeated intrathecal injection of AG14361 (25 μg/10 μL) reversed the increased protein expression of PARP‐1 in the DRGs and spinal cord on day 14 after paclitaxel injection (Figure 5F–H).

### 3.5 Identification of SIRT3 as a target through which PARP‐1 overactivation regulated the imbalance in mitochondrial oxidative metabolism in the PINP rats

**FIGURE 6:**
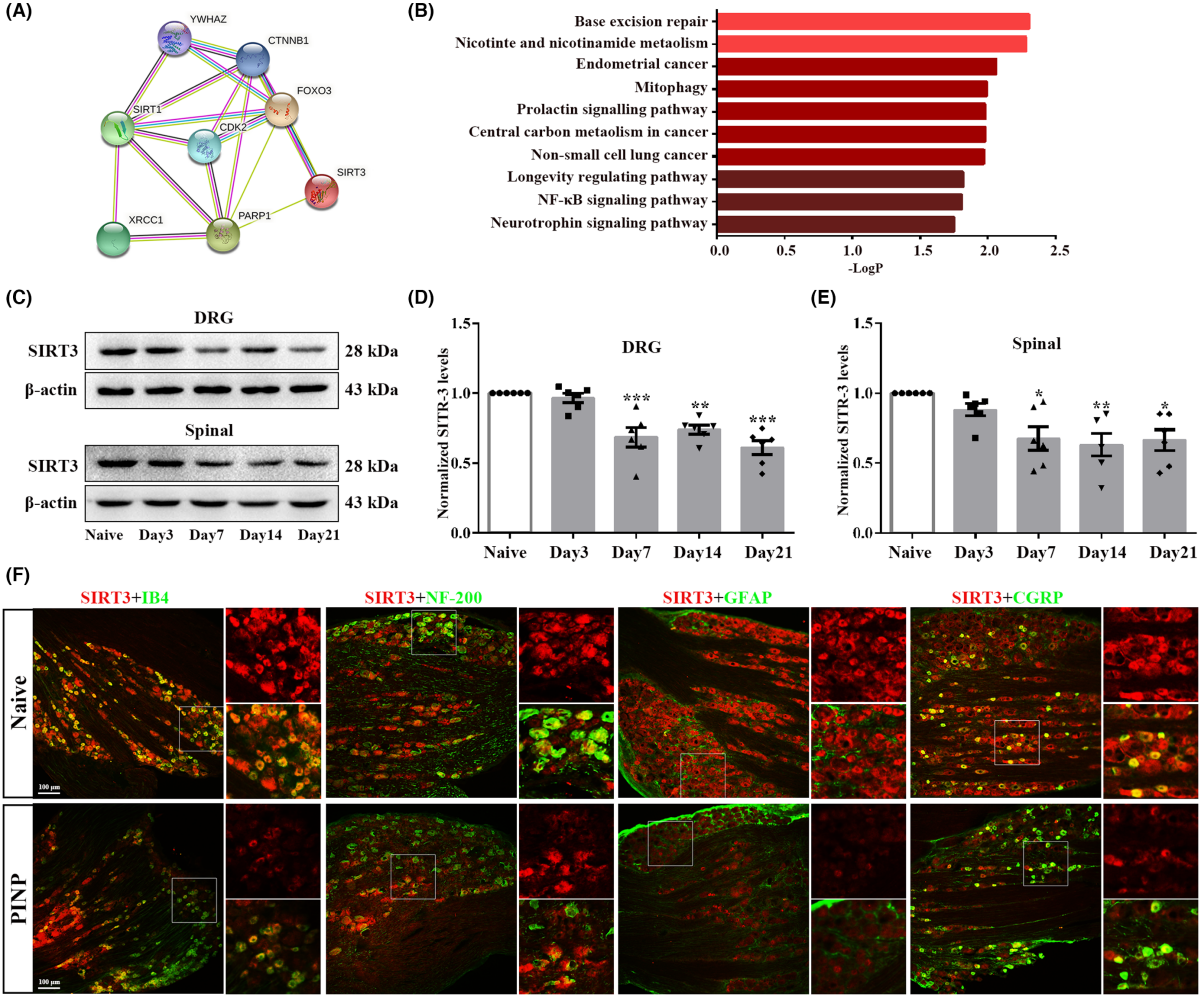
Identification of SIRT3 as a target through which PARP‐1 overactivation regulated the imbalance in mitochondrial oxidative metabolism in the PINP rats. (A, B) PPI analysis and KEGG pathways demonstrated an interaction network between PARP‐1 and SIRT3 along with the related signaling pathways. Species origin: Homo sapiens. Yellow line: Textmining. Blue line: From curated databases. purple line: Experimentally determined. black line: Co‐expression. (C–E) The levels of SIRT3 in the DRGs and spinal cord were decreased from day 7 to day 21 following paclitaxel injection. One‐way ANOVA followed by Bonferroni post hoc test, *p < 0.05, **p < 0.01, ***p < 0.001 versus Naïve group, *n* = 6 per group. (F) Representative double‐immunofluorescence staining of SIRT3 and IB4, NF‐200, CGRP or GFAP in the DRGs in each group. Scale bar: 100 μm, *n* = 3 per group.

SIRT3 is a conserved NAD^+^‐dependent deacetylase, and its downregulation has been shown to be associated with abnormal oxidative metabolism.^41^ Therefore, there is strong interest in further exploring whether PARP‐1 overactivation regulates mitochondrial oxidative metabolism by affecting SIRT3 activity in the PINP rats. First, the PPI network of STRING database demonstrated an interaction between PARP‐1 and SIRT3 (Figure 6A). The results related to signaling pathways revealed that these molecules are associated with various signaling pathways (Figure 6B). Then, we used western blot to analyze SIRT3 expression in each group. The protein levels of SIRT3 were markedly downregulated in the DRGs and spinal cord from day 7 to day 21 after paclitaxel injection (Figure 6C–E). Moreover, the cell types that expressed SIRT3 were consistent with those that expressed PARP‐1. SIRT3 was mainly colocalized with IB4‐labeled nonpeptidergic neurons, NF‐200‐labeled neurons, and CGRP‐labeled peptidergic neurons, but not with GFAP‐labeled satellite glial cells (Figure 6F). All these results suggested that PARP‐1 overactivation may regulate mitochondrial oxidative metabolism by affecting SIRT3 activity in the PINP rats.

### 3.6 Effect of paclitaxel injection on the deacetylase activity of SIRT3 in the DRGs and spinal cord of rats

**FIGURE 7:**
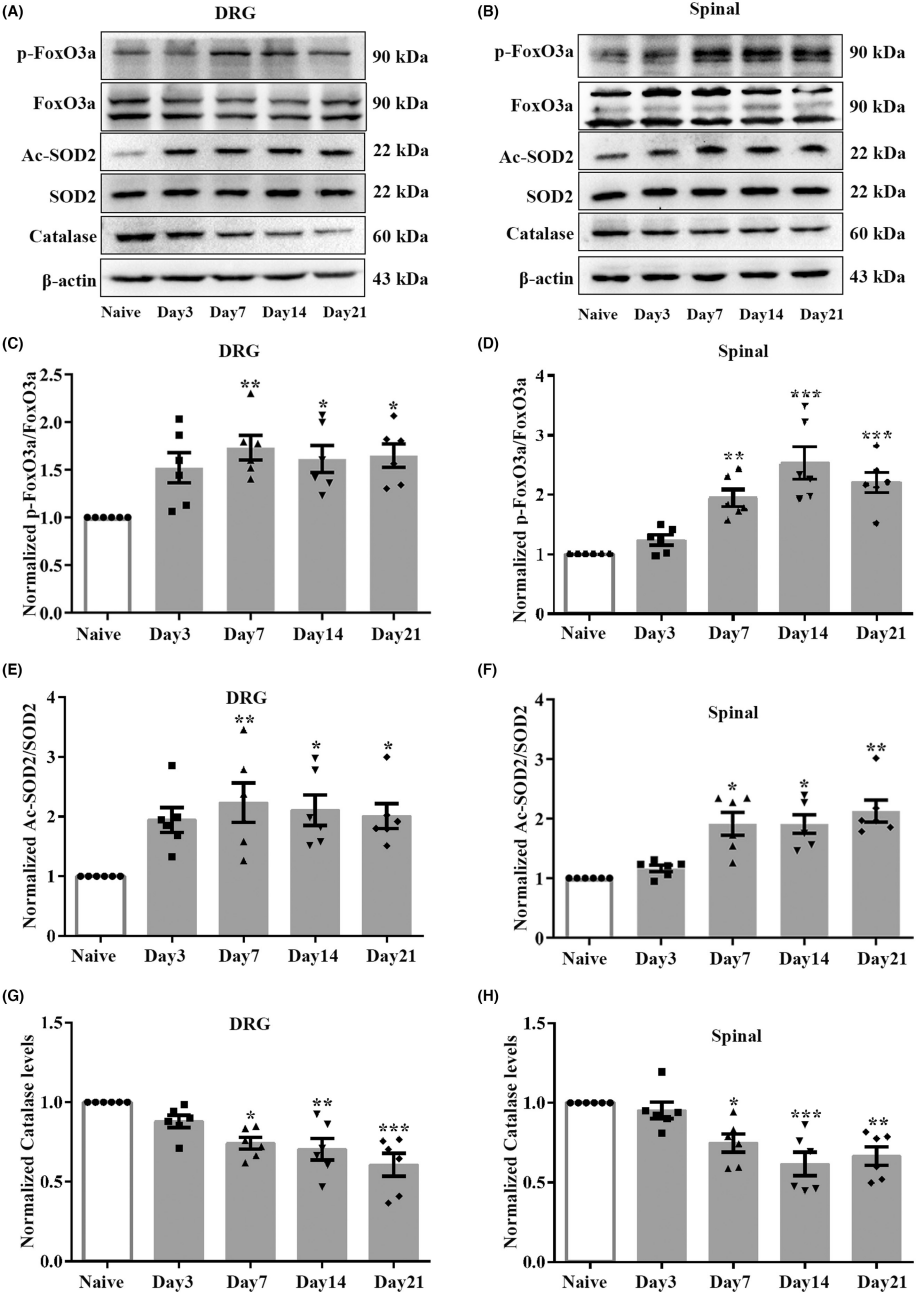
Effect of paclitaxel injection on deacetylase activity of SIRT3 in the DRGs and spinal cord in rats. (A–D) The ratios of p‐FoxO3a/FoxO3a were significantly upregulated in the DRGs and spinal cord after paclitaxel injection. One‐way ANOVA followed by Bonferroni post hoc test, *p < 0.05, **p < 0.01, ***p < 0.001 versus Naïve group, *n* = 6 per group. (E, F) The ratios of Ac‐SOD2/SOD2 were upregulated in the DRGs and spinal cord after paclitaxel injection. One‐way ANOVA followed by Bonferroni post hoc test was used for DRGs Ac‐SOD2/SOD2, Kruskal‐Wallis test followed by Dunn's multiple comparisons test was used for spinal Ac‐SOD2/SOD2, *p < 0.05, **p < 0.01 versus Naïve group, *n* = 6 per group. (G, H) The level of catalase in the DRGs and spinal cord was downregulated from day 7 to day 21 following paclitaxel injection. One‐way ANOVA followed by Bonferroni post hoc test, *p < 0.05, **p < 0.01, ***p < 0.001 versus Naïve group, *n* = 6 per group.

SIRT3 activity was proven to inhibit oxidative stress by directly regulating the deacetylation of FoxO3a and SOD2.^42^ Moreover, deacetylated FoxO3a further suppresses FoxO3a phosphorylation, thus enhancing the transcription of FoxO3a‐dependent antioxidant genes.^43^ To further substantiate the effect of paclitaxel injection on the deacetylase activity of SIRT3, we used western blot to examine the level changes of p‐FoxO3a, FoxO3a, Ac‐SOD2, as well as SIRT3‐related antioxidant genes, including catalase and SOD2 in the DRGs and spinal cord of rats in each group. The ratios of p‐FoxO3a/FoxO3a were increased in the DRGs and spinal cord after paclitaxel injection (Figure 7A–D). As shown in Figure 7G,H decreased levels of catalase were observed in the DRGs and spinal cord in the PINP group compared to the Naïve group. In addition, the ratios of Ac‐SOD2/SOD2 were markedly upregulated in the DRGs and spinal cord in the PINP group (Figure 7E,F). Together, these results suggested decreased expression and deacetylase activity of SIRT3 in the DRGs and spinal cord of rats after paclitaxel injection.

### 3.7 Specific PARP‐1 inhibition enhanced oxidative metabolism by regulating the expression and deacetylase activity of SIRT3 in the PINP rats

**FIGURE 8:**
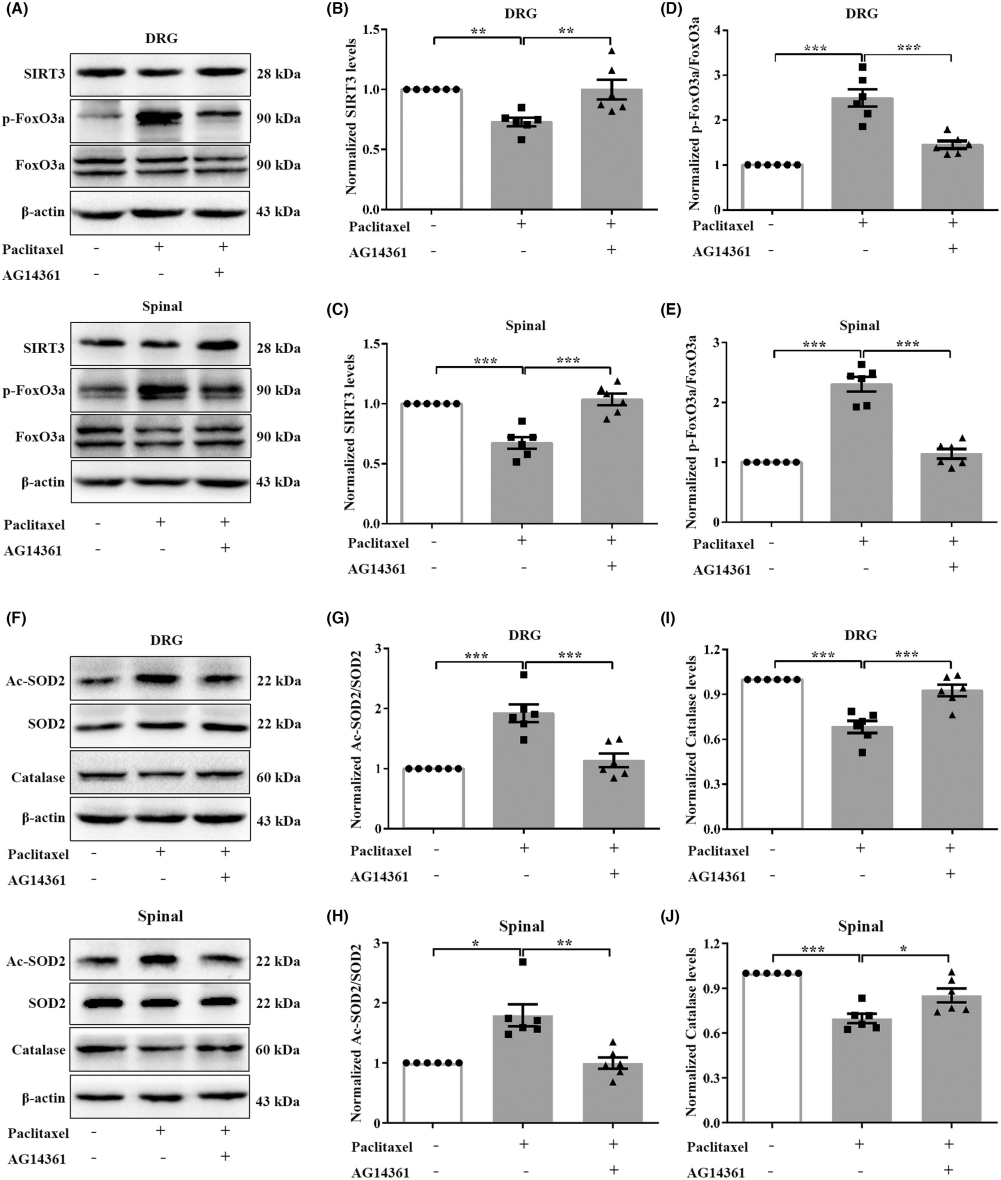
Specific PARP‐1 inhibition enhanced oxidative metabolism by upregulating the expression and deacetylase activity of SIRT3 in the PINP rats. (A–C) Specific PARP‐1 inhibitor AG14361 administration reversed the decreased expressions of SIRT3 in the DRGs and spinal cord in the PINP group. One‐way ANOVA followed by Bonferroni post hoc test, **p < 0.01, ***p < 0.001 versus corresponding groups, *n* = 6 per group. (D–H) Intrathecal administration of AG14361 significantly inhibited the increased ratios of p‐FoxO3a/FoxO3a and Ac‐SOD2/SOD2 in the DRGs and spinal cord in the PINP rats. One‐way ANOVA followed by Bonferroni post hoc test for p‐FoxO3a/FoxO3a and DRGs Ac‐SOD2/SOD2, Kruskal‐Wallis test followed by Dunn's multiple comparisons tests was used for spinal Ac‐SOD2/SOD2, *p < 0.05, **p < 0.01, ***p < 0.001 versus corresponding groups, *n* = 6 per group. (I–J) Upon AG14361 treatment, the decreased protein levels of catalase in the DRGs and spinal cord were significantly reversed in the PINP rats. One‐way ANOVA followed by Bonferroni post hoc test, *p < 0.05, ***p < 0.001 versus corresponding groups, *n* = 6 per group.

Further studies explored whether specific inhibition of PARP‐1 alleviated PINP‐induced oxidative stress by regulating the expression and deacetylase activity of SIRT3. As expected, treatment with specific PARP‐1 inhibitor AG14361 reversed the decreased expression of SIRT3 in the DRGs and spinal cord in the PINP rats (Figure 8A–C). Moreover, the intrathecal administration of AG14361 significantly inhibited the increase in p‐FoxO3a/FoxO3a and Ac‐SOD2/SOD2 ratios in the DRGs and spinal cord in the PINP rats (Figure 8D–H). We also assessed the levels of catalase in the DRGs and spinal cord of rats in each group. As expected, intrathecal administration of AG14361 significantly reversed the PINP‐induced decrease in the catalase levels in the DRGs and spinal cord of rats (Figure 8I,J).

### 3.8 Administration of NAD ^+^ precursor NMN protected against PINP‐induced oxidative stress by upregulating the deacetylase activity of SIRT3 in rats

**FIGURE 9:**
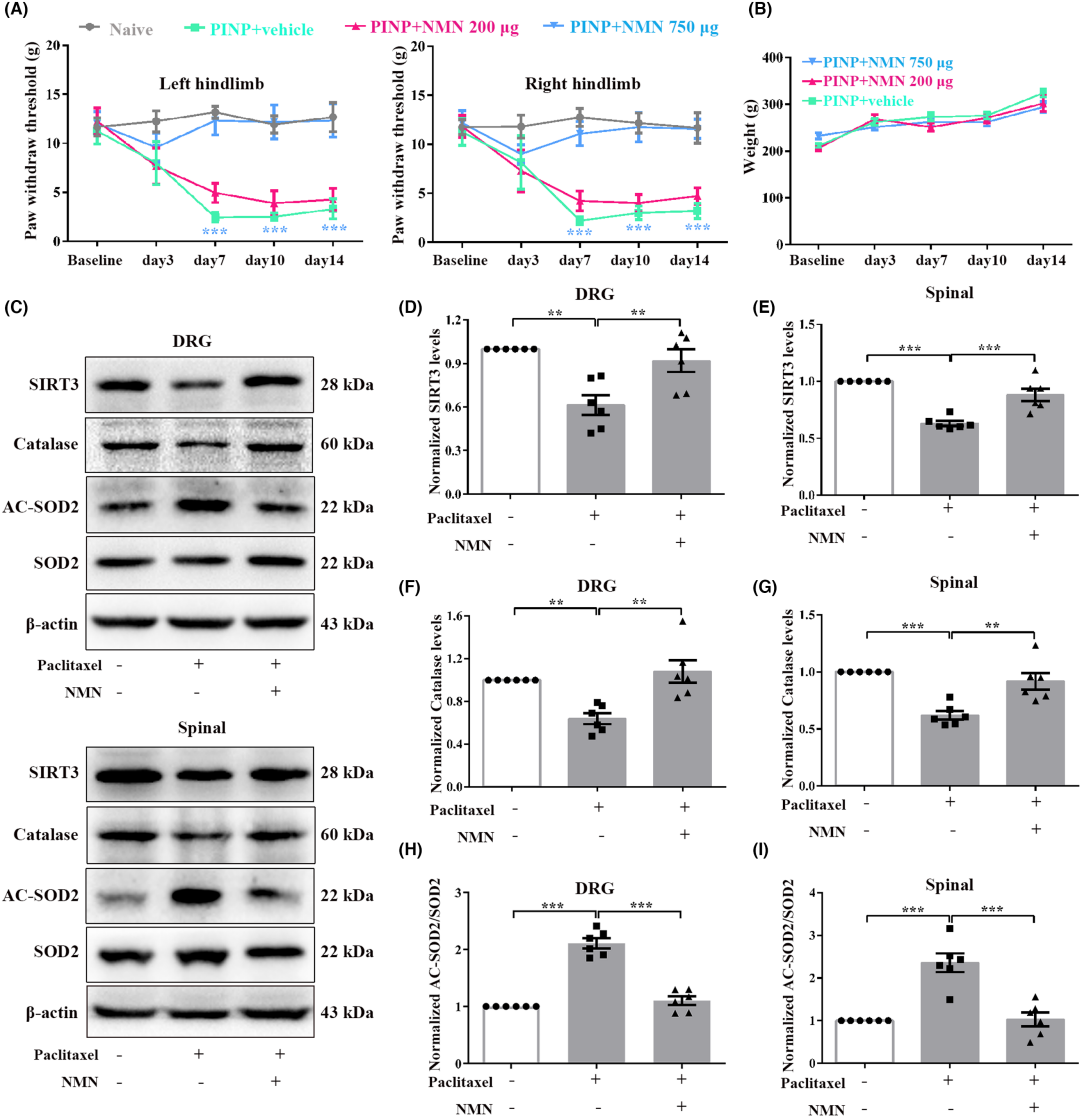
Replenishment of NAD^+^ precursor NMN reversed paclitaxel‐induced pain hypersensitivity. (A) Continuous intrathecal administration of NAD^+^ precursor NMN alleviated paclitaxel injection‐induced mechanical allodynia in a dose‐dependent manner. Two‐way ANOVA followed by Bonferroni post hoc test, ***p < 0.001 PINP+NMN 750 μg group versus PINP+vehicle group, *n* = 6 per group. (B) No significant differences of the body weight were observed in PINP+NMN groups compared to PINP+vehicle group. Two‐way ANOVA followed by Bonferroni post hoc test, *p* > 0.05, *n* = 6 per group. (C–I) PINP‐induced downregulations of SIRT3 and catalase protein levels and increased ratios of Ac‐SOD2/SOD2 in the DRGs and spinal cord of rats were significantly reversed by NMN (750 μg/15 μl) administration. One‐way ANOVA followed by Bonferroni post hoc test, **p < 0.01, ***p < 0.001 versus corresponding groups, *n* = 6 per group.

Bioinformatics analysis showed that PARP‐1 and SIRT3‐related antioxidative genes are critically related to nicotinate and nicotinamide metabolism. Moreover, we observed a significant depletion of NAD^+^ levels in the DRGs and spinal cord in the PINP rats. We hypothesized that the decreased NAD^+^ level may be a crucial mechanism by which PARP‐1 overactivation suppresses the activity of SIRT3 in the PINP rats. Next, we sought to analyze the effect of the NAD^+^ precursor NMN on paclitaxel injection‐induced pain hypersensitivity. As expected, the decrease in the PWTs was partially reversed by repeated intrathecal injection of NMN (750 μg/15 μL), but not NMN (200 μg/10 μL) (Figure 9A). In addition, no significant differences in body weight were observed in the PINP+NMN groups compared to the PINP+vehicle group (Figure 9B). We further explored whether intrathecal injection of NMN affected the activity of SIRT3 in the PINP rats. As shown in Figure 9C–G, PINP‐mediated downregulation of SIRT3 and catalase were reversed by NMN administration (Figure 9C–G). In addition, intrathecal injection of NMN (750 μg/15 μL) abrogated increase in the ratios of Ac‐SOD2/SOD2 in the DRGs and spinal cord in the PINP rats (Figure 9H,I). Collectively, these results suggested that NMN administration prevented PINP‐induced imbalance in oxidative metabolism by increasing the expression and deacetylase activity of SIRT3.

### 3.9 SIRT3 activity is critical for the analgesic effect of specific PARP‐1 inhibition in the PINP rats

**FIGURE 10:**
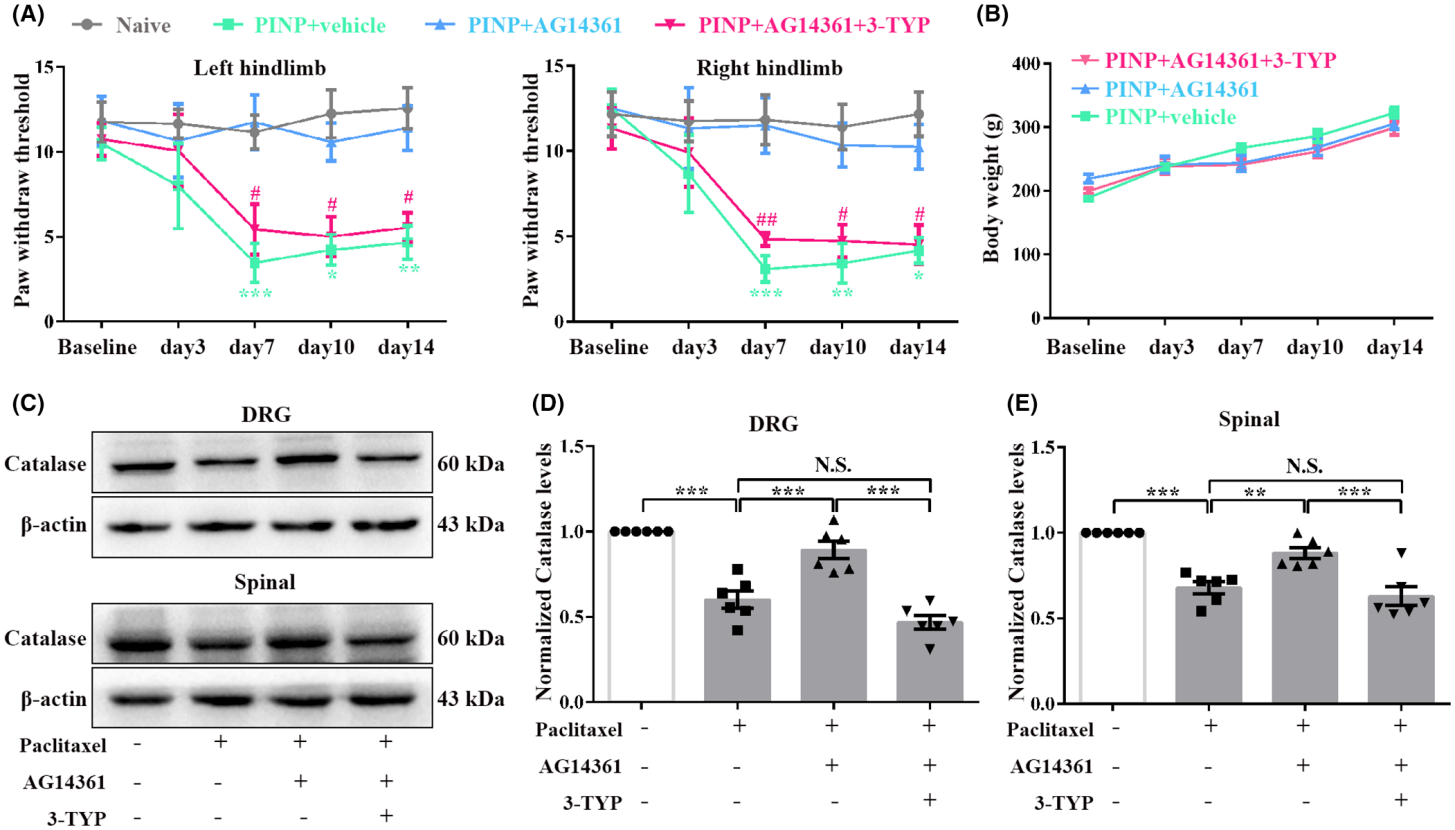
SIRT3 activity is critical for the analgesic effect of specific PARP‐1 inhibition in the PINP rats. (A, B) Continuous intrathecal injection of AG14361 significantly reversed the decreased PWTs in the PINP rats. However, pre‐injection of SIRT3 inhibitor 3‐TYP (150 μg/15 μL) 30 min before AG14361 (25 μg/10 μL) partly suppressed the analgesic effect of AG14361 in the PINP rats. Two‐way ANOVA followed by Bonferroni post hoc test, *p < 0.05, **p < 0.01, ***p < 0.001 PINP+AG14361 group versus PINP+vehicle group, #*p* < 0.05, ##*p* < 0.01 PINP+AG14361+3‐TYP group versus PINP+AG14361 group, *n* = 6 per group. (C) No significant differences of the body weight were observed in PINP+AG14361+3‐TYP group compared to PINP+vehicle group. Two‐way ANOVA followed by Bonferroni post hoc test, *p* > 0.05, *n* = 5–6 per group. (D–F) The increased levels of catalase in the DRGs and spinal cord caused by AG14361 (25 μg/10 μL) treatment were partly reversed by 3‐TYP (150 μg/15 μL) injection in the PINP rats. One‐way ANOVA followed by Bonferroni post hoc test, **p < 0.01, ***p < 0.001 versus corresponding groups, *n* = 6 per group.

We next explored whether pre‐injection of SIRT3 inhibitor 3‐TYP reversed the analgesic effect of AG14361 in the PINP rats. As expected, pre‐injection of 3‐TYP (150 μg/15 μL) 30 min before AG14361 (25 μg/10 μL) partly downregulated the increased PWTs in the PINP rats (Figure 10A). No significant differences of the body weight were observed in PINP+AG14361+3‐TYP group compared to PINP+vehicle group (Figure 10B). Furthermore, the increased levels of catalase caused by AG14361 were partly reversed by 3‐TYP injection in the DRGs and spinal cord in the PINP rats (Figure 10C–E). These findings further confirmed that PARP‐1 overactivation, accompanied by the depletion of NAD^+^, thus affects the expressions and deacetylase activities of SIRT3 in the DRGs and spinal cord in the PINP rats.

## 4 DISCUSSION

In the current study, we verified that DNA oxidative damage‐induced PARP overactivation resulted in NAD^+^ depletion, and then impedes cellular energy metabolism in the PINP rats. This finding suggested excessive PARP activation as a crucial process in the pathogenesis of PINP. Therefore, we developed an Olaparib PROTAC to achieve the efficient degradation of PARP and further explored the analgesic effect of this PROTAC in the PINP rats. Continuous intrathecal injection of Olaparib PROTAC protected against paclitaxel injection‐induced mechanical allodynia in rats. Moreover, no significant motor function impairment or organ toxicity was observed after Olaparib PROTAC administration. PARP‐1, but not PARP‐2, is proved to be a crucial molecule involved in the development of PINP. Therefore, further studies elucidated that specific inhibition of PARP‐1 enhanced redox metabolism partly by increasing the expression and deacetylase activity of SIRT3 in the PINP rats. Moreover, exogenous replenishment of NAD^+^ precursor NMN reversed paclitaxel‐induced pain hyperalgesia and the decreased expression and deacetylase activity of SIRT3, suggesting that increasing NAD^+^ levels may be a crucial mechanism by which PARP‐1 inhibition activates SIRT3 in the PINP rats.

Previous studies have revealed that mild stress promotes PARP‐1 activation to initiate DNA repair, without consuming NAD^+^ levels. While excessive PARP‐1 activation caused NAD^+^ depletion, thus resulting in the cellular energy depletion.^16^, ^44^ Consistent with these studies, we reported a remarkable increase in PARP‐1 protein level as well as NAD^+^ and ATP depletions in the DRGs and spinal cord in the PINP rats, suggesting that PARP‐1 overactivation is a crucial process in the pathogenesis of PINP. NAD^+^ is a co‐substrate for SIRTs in the catalysis of target protein deacetylation.^45^, ^46^ The ability of PARP‐1 overactivation to decrease ‐ NAD^+^ levels prompted us to further explore whether PARP‐1 overactivation suppressed oxidative metabolism by downregulating SIRTs activity in the PINP rats. Recent studies have supported an interaction between SIRT3 and PARP‐1 in chronic disorders, such as cardiac hypertrophy and diabetic retinopathy.^47^, ^48^ Our western blot results revealed decreased expression and deacetylase activity of SIRT3 in the PINP rats. Moreover, the PPI network demonstrated an interaction between PARP‐1 and SIRT3, suggesting that PARP‐1 overactivation may regulate mitochondrial oxidative metabolism by affecting SIRT3 activity. Interestingly, further results suggested that specific inhibition of PARP‐1 protected against PINP by upregulating the expression and deacetylase activity of SIRT3 in the PINP rats. As previous studies showed, when severe DNA disruption occurs, excessively activated PARP‐1 accelerates the PAR polymers accumulation and persistent consumption of large amounts of NAD^+^ and ATP, which further induces parthanatos, a type of PARP‐1‐dependent cell death initiated by PARP‐1 hyperactivation.^49^, ^50^ In our study, we observed that overactivation of PARP‐1 directly led to a significant decrease in the levels of NAD^+^ in the DRGs and spinal cord in PINP rats. Therefore, we only focused on the effect of NAD^+^ content on the expression of SIRT3. Given that PARP‐1 overactivation is involved in cell death. Whether PARP‐1 inhibition regulates SIRT3 expression by affecting cell death remains to be further explored. Moreover, exogenous replenishment of the NAD^+^ precursor NMN reversed paclitaxel‐induced hyperalgesia and decreased SIRT3 deacetylase activity in rats. All these results suggested that the decreasing NAD^+^ levels may be a crucial mechanism by which PARP‐1 overactivation suppresses the activity of SIRT3 in the PINP rats. However, previous research revealed that NAD^+^ is membrane impermeable, and the NAD^+^‐dependent signaling axis is compartmentalized within the cell.^14^, ^51^ Interestingly, recent studies suggested the occurrence of NAD^+^‐dependent crosstalk in compartmentalized organelles.^52^, ^53^ Hopp et al. proposed that NAD^+^ in the mitochondria could be released to sustain nuclear PARP‐1‐induced ADP‐ribosylation in response to stress, suggesting a NAD^+^‐dependent mitochondrial‐nuclear crosstalk.^53^ However, we cannot exclude the possibility that PARP‐1 inhibition impacts SIRT3 through other mechanisms, possibly via reciprocal transcriptional or posttranscriptional modification. Further studies are encouraged to elucidate whether there are other potential mechanisms by which PARP‐1 inhibition regulates SIRT3 activity.

In summary, our study verified the induction of DNA damage, PARP‐1 overactivation, and subsequent NAD^+^ depletion as crucial events in the development of PINP. Importantly, specific inhibition of PARP‐1 enhanced redox metabolism partly by increasing the expression and deacetylase activity of SIRT3 in the DRGs and spinal cord in the PINP rats. In addition, increasing the levels of NAD^+^ may be a crucial mechanism by which PARP‐1 inhibition enhances the activity of SIRT3. Our study provides a novel insight into the mechanism of DNA oxidative damage in the development of PINP and implicates PARP‐1 as a possible therapeutic target for clinical PINP treatment.

## FUNDING INFORMATION

This work was supported by the National Natural Science Foundation of China (No 81873732).

## CONFLICT OF INTEREST STATEMENT

The authors have declared that no conflict of interest exists.

## Supporting information


Data S1.

## Data Availability

The data that support the findings of this study are available from the corresponding author upon reasonable request.
