# Supplementary material for: DNA damage induced PARP‐1 overactivation confers paclitaxel‐induced neuropathic pain by regulating mitochondrial oxidative metabolism
Source: CNS Neurosci Ther. 2024 Aug 30;30(9):e70012. doi: 10.1111/cns.70012 (PMC11364515; doi:10.1111/cns.70012)
Supplement: Supplementary file 1 — Data S1. [file CNS-30-e70012-s001.zip › Supplemental data revision.docx]

**DNA damage induced PARP-1 overactivation confers paclitaxel-induced neuropathic pain by regulating mitochondrial oxidative metabolism**

**Supplemental method 1. The specific synthesis process of Olaparib PROTAC.**

100 ml reaction tube was charged with compound **1** (0.366 g, 1.0 mmol, 1.0 equivalent) and 1 equivalent of pent-4-ynoic acid **2**. Then 25 mL dry dichloromethane was added. The reaction was stirred at 0 ℃, then 1.2 equivalents of EDCI, HOBt and DIPEA was added in sequence. Then the reaction was warmed to room temperature (RT) and stirred overnight. The reaction was monitored by TLC. When the reaction completed, 25 mL dichloromethane and 25 mL saturated NaHCO_3_ aqueous was added. The organic phase was separated and washed by 25 mL brine and then dried over Na_2_SO_4_. The solvent was removed with rotary evaporator. The residue was purified with column chromatography on silica gel, eluting with dichloromethane and methanol to afford the corresponding product **3** as white solid, 0.339 g, 76% yield.

25 ml reaction tube was charged with compound **4** (0.27 g, 1.0 mmol, 1.0 equivalent) and 1 equivalent of 2-(2-azidoethoxy) ethanamine **5**. Then 5 mL dry DMF was added. 2 equivalents of DIPEA were added to the mixture and the resulting mixture was stirred at 80 ℃ overnight. The reaction was monitored by TLC. When the reaction completed, 30 mL ethyl acetate was added and the mixture was washed by brine for several times and then dried over Na_2_SO_4_. The solvent was removed and the residue was purified with column chromatography on silica gel, eluting with petroleum ether and ethyl acetate to afford the corresponding product **6** as yellow solid, 0.116 g, 30% yield.

5 ml reaction tube was charged with alkyne **3** (0.089 g, 0.2 mmol, 1.0 equivalent) and 1 equivalent of azide **6**. Then 2 mL THF was added. The reaction was stirred at RT. 1 equivalent of CuSO_4_ and 2 equivalents of Sodium Ascorbate was dissolved in 0.5 mL water and the solution color became brown. The brown aqueous was added in dropwise to the THF solution. Then the resulted mixture was stirred at RT for 2 hours. The reaction was monitored by TLC. When the reaction completed, 10 mL ethyl acetate and 10 mL brine was added. The organic phase was separated and dried over Na_2_SO_4_. The solvent was removed with rotary evaporator. The residue was purified with column chromatography on silica gel, eluting with dichloromethane and methanol to afford the corresponding Olaparib PROTAC product as yellow solid, 0.058 g, 35% yield.

**Supplemental table 1. The reagents or resources used in this study.**

| Reagents or resources | **Source** | **Identifier** |
| --- | --- | --- |
| Antibodies |  |  |
| Rabbit polyclonal anti-PARP-1 | ABclonal | Cat #A0942; RRID: AB_2757470 |
|  | Cell Signaling Technology | Cat #9532; RRID: AB_659884 |
| Rabbit polyclonal anti-PARP-2 | ABclonal | Cat #A16475; RRID: AB_2770793 |
| Rabbit monoclonal anti-Phospho-γH2A.X (Ser 139) | Cell Signaling Technology | Cat #9718; RRID: AB_2118009 |
| Mouse monoclonal anti-Phospho-γH2A.X (Ser 139) | Abcam | Cat #ab26350; RRID: AB_470861 |
| Rabbit monoclonal anti-β-actin | ABclonal | Cat #AC026; RRID: AB_2768234 |
| Rabbit monoclonal anti-SIRT3 | Cell Signaling Technology | Cat #2627; RRID: AB_2188622 |
|  | Affinity Biosciences | Cat #AF5135; RRID: AB_2837035 |
| Rabbit polyclonal anti-Catalase | ABclonal | Cat #A11777 |
| Rabbit polyclonal anti- manganese superoxide dismutase (SOD2) | ABclonal | Cat #A1340; RRID: AB_2760260 |
| Rabbit polyclonal anti-[Acetyl-SOD2/MnSOD (Lys68)](http://www.affbiotech.cn/goods-17426-AF3751-Acetyl-SOD2_MnSOD+%28Lys68%29+Antibody.html) | Affinity Biosciences | Cat #AF3751; RRID: AB_2847065 |
| Rabbit polyclonal anti-phospho-FoxO3a-S253 | ABclonal | Cat #AP0684; RRID: AB_2771125 |
|  | Affinity Biosciences | Cat # [AF3020](http://www.affbiotech.cn/goods-1173-AF3020-Phospho_FOXO3A_Ser253_Antibody.html); RRID: AB_2834427 |
| Rabbit polyclonal anti-FoxO3a | ABclonal | Cat #A0120; RRID: AB_2771126 |
| HRP-conjugated goat anti-rabbit IgG | Promotor |  |
| Mouse anti-[8-hydroxy-2'-deoxyguanosine](https://www.abcam.cn/8-hydroxy-2-deoxyguanosine-8-ohdg-elisa-kit-ab285254.html) (8-OHdG) | Abcam | Cat #ab48508; RRID: AB_867461 |
| Mouse anti-calcitonin gene related peptide (CGRP) | Abcam | Cat #ab81887; RRID: AB_1658411 |
| Mouse anti-neurofilament-200 (NF-200) | Abcam | Cat #ab134306; RRID: AB_2895532 |
| BS-Isolectin B4 FITC Conjugate (IB4) | Sigma-Aldrich | Cat #2895; |
| Mouse anti-glial fibrillary acidic protein (GFAP) | Cell Signaling Technology | Cat #34001; RRID: AB_2799042 |
| 488-conjugated goat anti-mouse IgG | Proteintech | Cat #SA00013-1; RRID: AB_2810983 |
| cy3-conjugated goat anti-rabbit IgG | Proteintech | Cat #SA00013-4; RRID: AB_2810984 |
| FITC-conjugated donkey anti-goat IgG | Proteintech | Cat #SA00003-4; RRID: AB_2890898 |
| cy3-conjugated donkey anti-rabbit IgG | Proteintech | Cat #SA00013-8; RRID: AB_2857367 |
| Chemicals, peptides, and recombinant proteins | | |
| Paclitaxel | MedChemExpress | Cat #HY-B0015/CS-1145 |
| PARP-1 inhibitor AG14361 | MedChemExpress | Cat #HY-12032 |
| Olaparib PROTAC |  | This paper |
| NAD^+^ precursor NMN | MedChemExpress | Cat #HY-F0004/CS-4996 |
| SIRT3 inhibitor 3-TYP | MedChemExpress | Cat #HY-108331/CS7841 |
| Phosphate buffered saline (PBS) | Servicebio | Cat #G4202 |
| Isoflurane | RWD Life Science | Cat #R510-22 |
| Dimethyl sulfoxide (DMSO) | Boster | Cat #1084ML100 |
| PEG300 | Selleck | Cat #S6704 |
| Tween 80 | Boster | Cat #1716ML100 |
| Protease [inhibitor](javascript:;) | Boster | Cat #AR1182 |
| Phosphatase [inhibitor](javascript:;) | Boster | Cat #AR1183 |
| Radioimmunoprecipitation assay buffer | Boster | Cat #AR0102-100 |
| Ultra high sensitivity ECL Kit | MedChemExpress | Cat #HY-K1005 |
| Normal goat serum (10%) | Boster | Cat #AR0009 |
| Normal donkey serum (10%) | Boster | Cat #ANT051 |
| Triton X-100 (0.3%) | Boster | Cat #1139ML100 |
| 4,6-diamidino-2-phenylindole (DAPI) | Boster | Cat #AR1176 |
| Epoxy resin monomer | SPI-CHEM | Cat #90529-77-4 |
| Glutaraldehyde (2.5%) | Alfa Aesar | Cat #17876 |
| Critical commercial kit |  |  |
| Bicinchoninic acid (BCA) protein assay kit | Boster | Cat #AR0146 |
| [Malondialdehyde](http://www.njjcbio.com/products.asp?id=287) (MDA) Assay Kit | Nanjing Jiancheng Bio-Technology | Cat #A003-1 |
| ROS Assay Kit | Nanjing Jiancheng Bio-Technology | Cat #E004-1-1 |
| Adenosine triphosphate (ATP) Assay Kit | Nanjing Jiancheng Bio-Technology | Cat #A095 |
| NAD^+^/NADH Assay Kit | Beyotime Biotechnology | Cat #S0175 |

**Supplemental table 2. The gait scores of rats during spontaneous ambulation in each group.**

| **Group** | **Levels of motor function** | | | |
| --- | --- | --- | --- | --- |
|  | Ⅰ | Ⅱ | Ⅲ | Ⅳ |
| **PINP+vehicle** | 13/15 | 2/15 | 0/15 | 0/15 |
| **PINP+Olaparib PROTAC** | 12/15 | 2/15 | 1/15 | 0/15 |


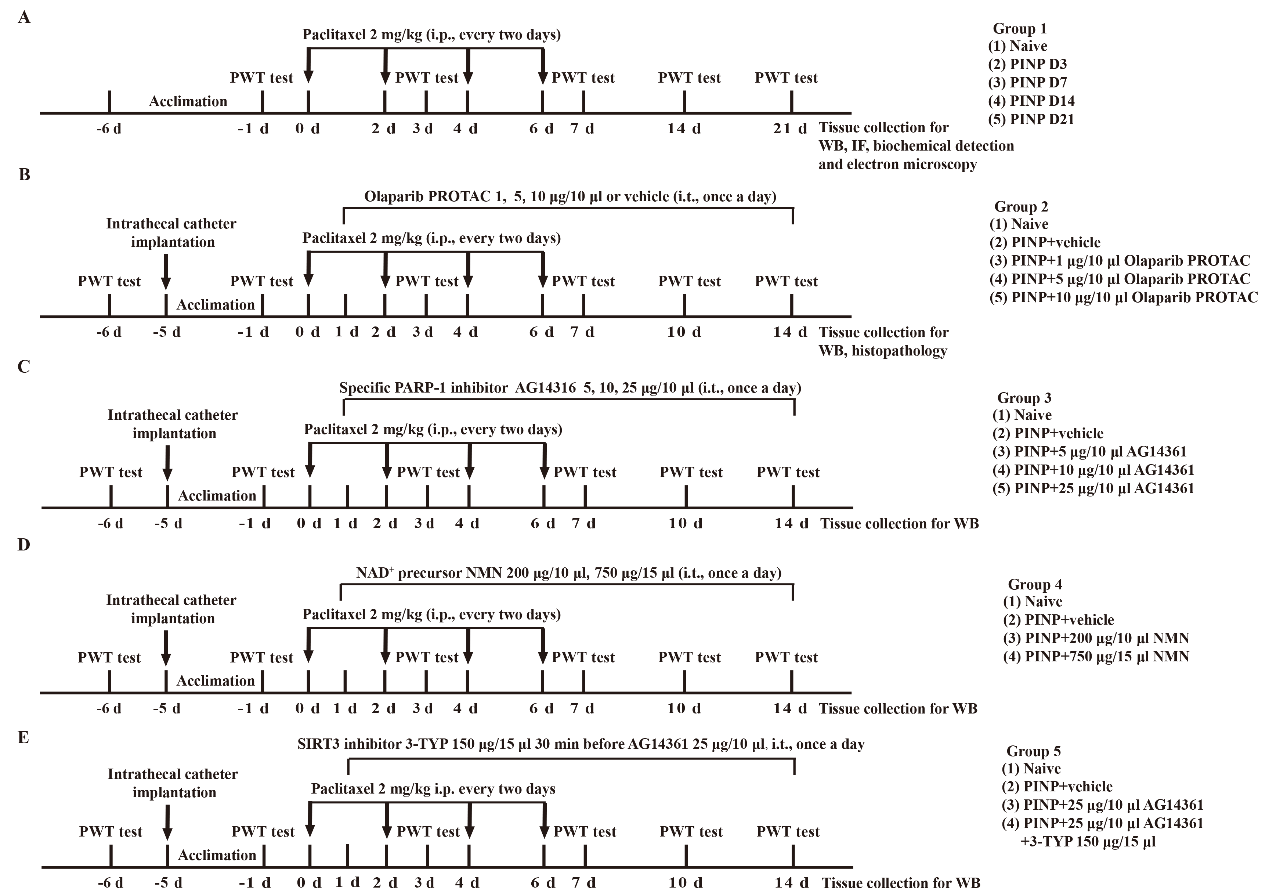


**Figure S1. Schematic illustration of the experimental designs and animal groups.** (A) Changes in pain behavior and PARP expression after paclitaxel injection. (B) Effect of continuous intrathecal injection of Olaparib PROTAC on paclitaxel-induced mechanical allodynia in the PINP rats. (C) Effects of continuous intrathecal administration of specific PARP-1 inhibitor AG14361 on paclitaxel-induced mechanical allodynia as well as expression and deacetylase activity of SIRT3 in the PINP rats. (D) Effects of continuous intrathecal injection of NAD^+^ precursor NMN on mechanical allodynia as well as expression and deacetylase activity of SIRT3 in the PINP rats. (E) Effect of pre-injection of SIRT3 inhibitor 3-TYP 30 min before AG14361 on paclitaxel-induced mechanical allodynia.
