# Supplementary figures and images for: DNA damage induced PARP‐1 overactivation confers paclitaxel‐induced neuropathic pain by regulating mitochondrial oxidative metabolism
Source: CNS Neurosci Ther. 2024 Aug 30;30(9):e70012. doi: 10.1111/cns.70012 (PMC11364515; doi:10.1111/cns.70012)

Figure 1

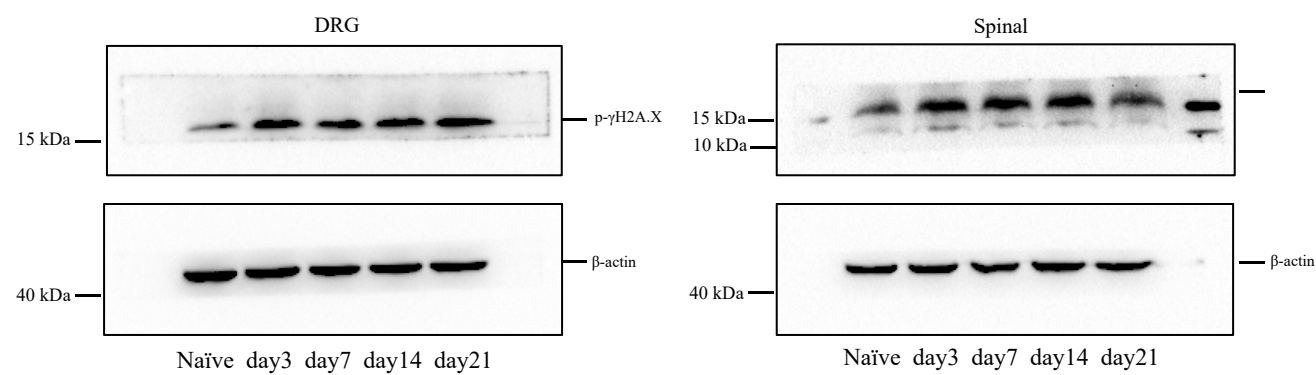

Figure 2

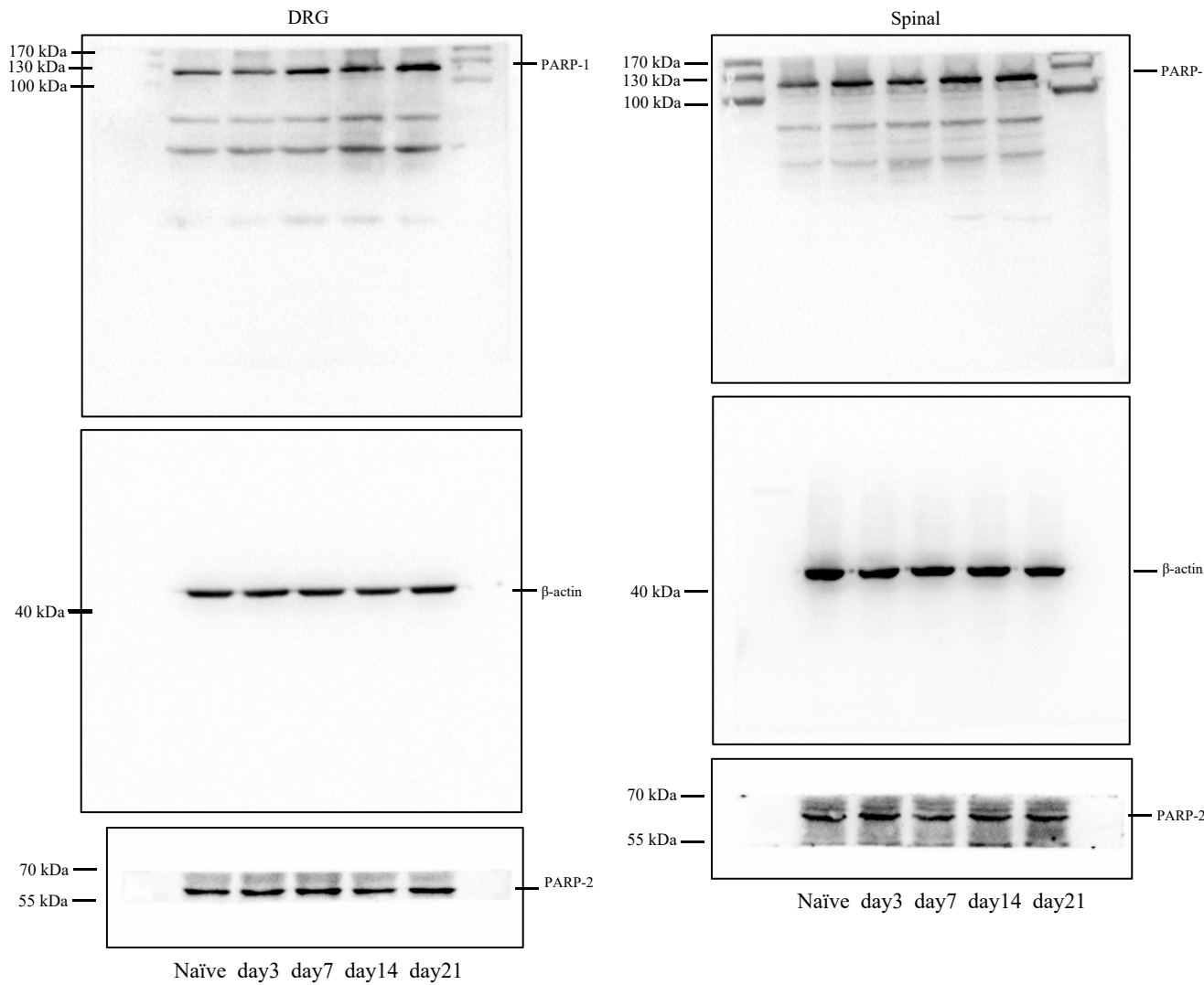

Figure 3

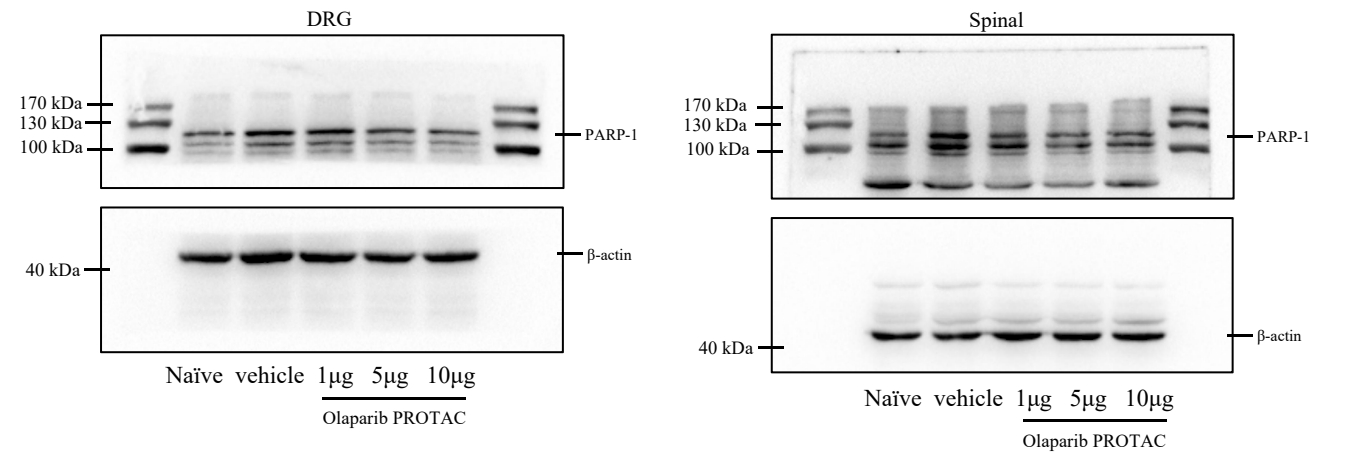

Figure 5

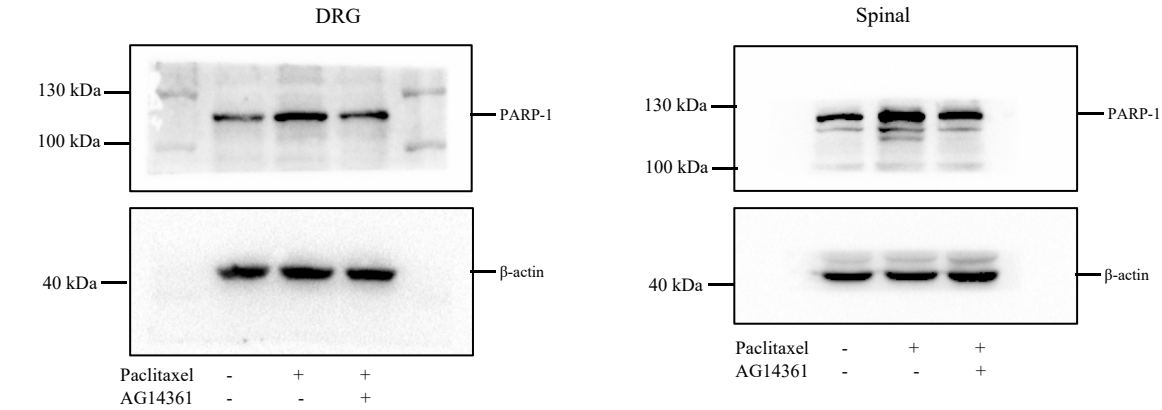

Figure 6

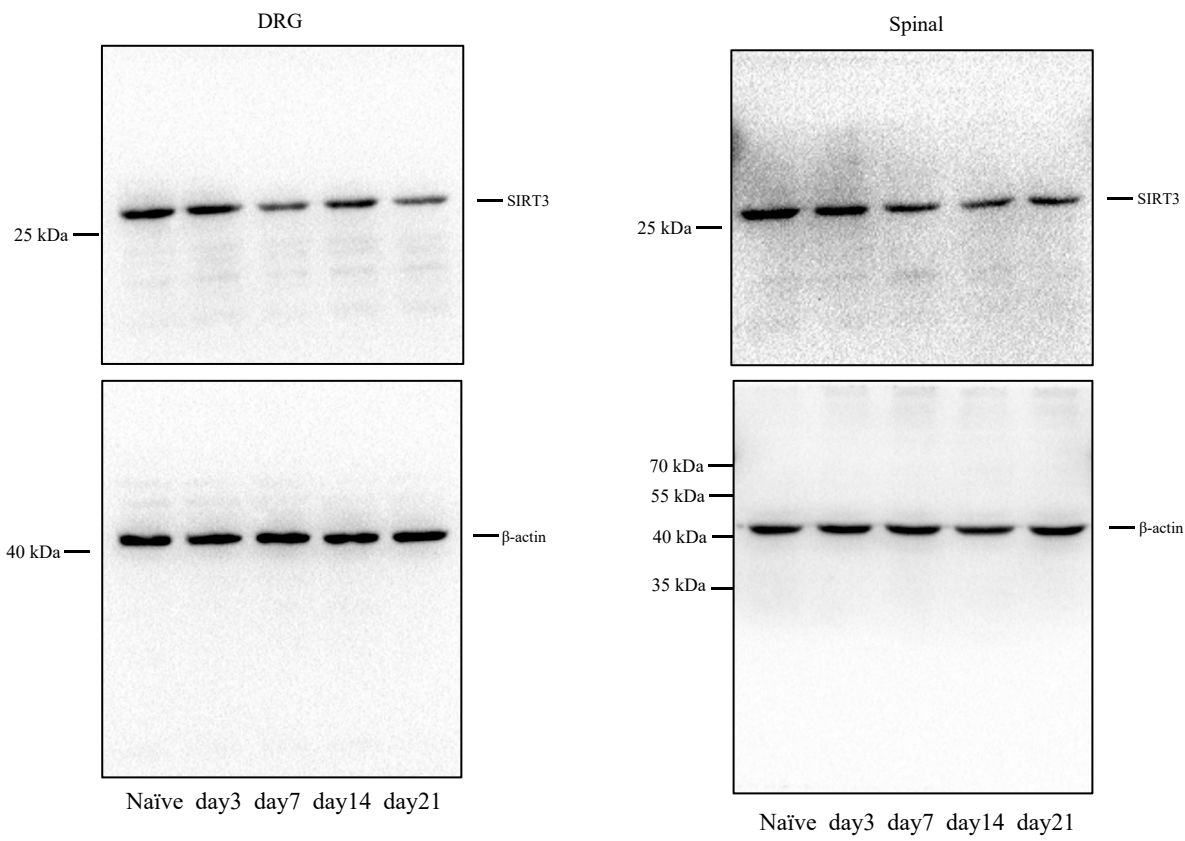

Figure 7

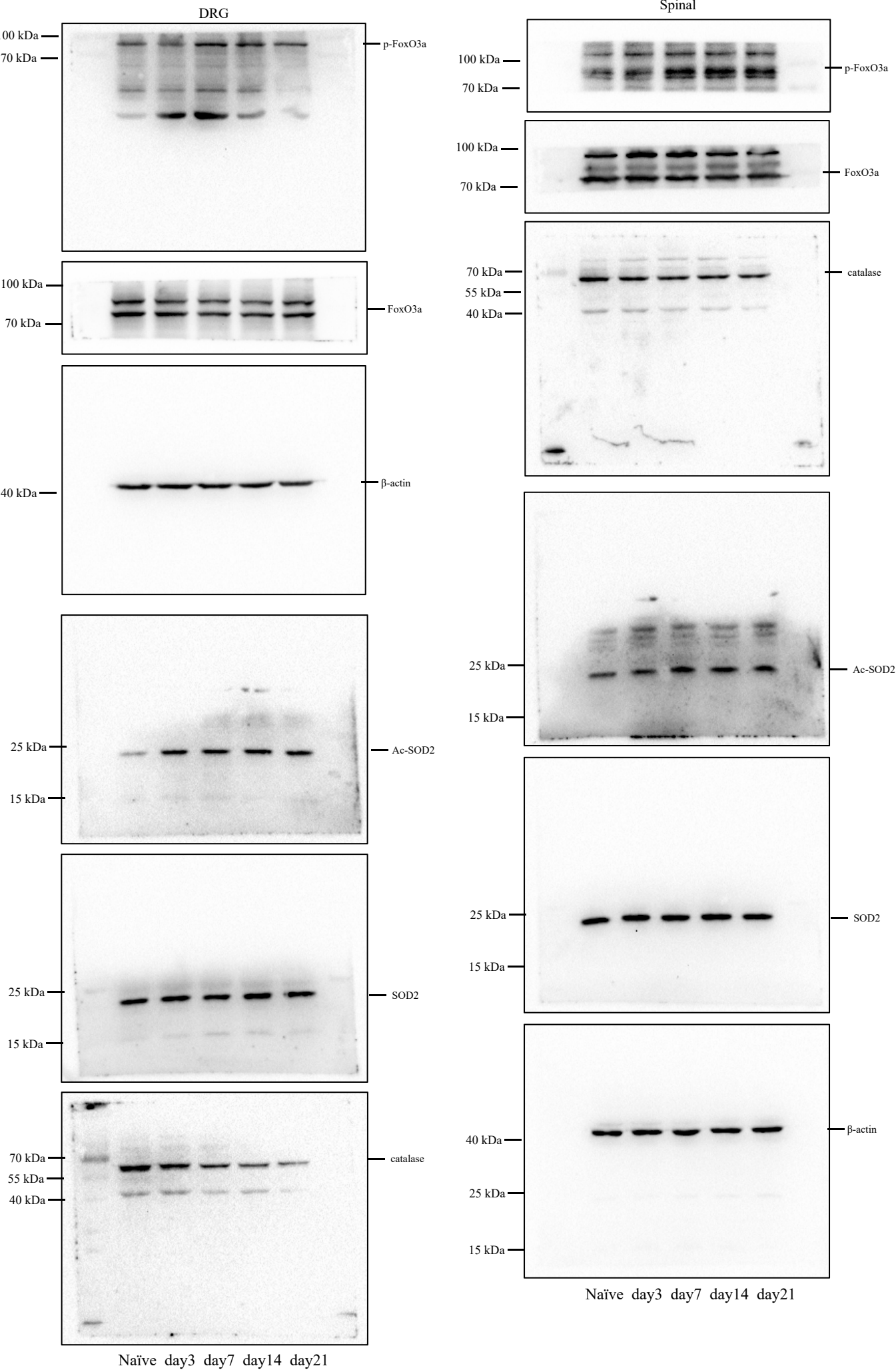

Figure 8

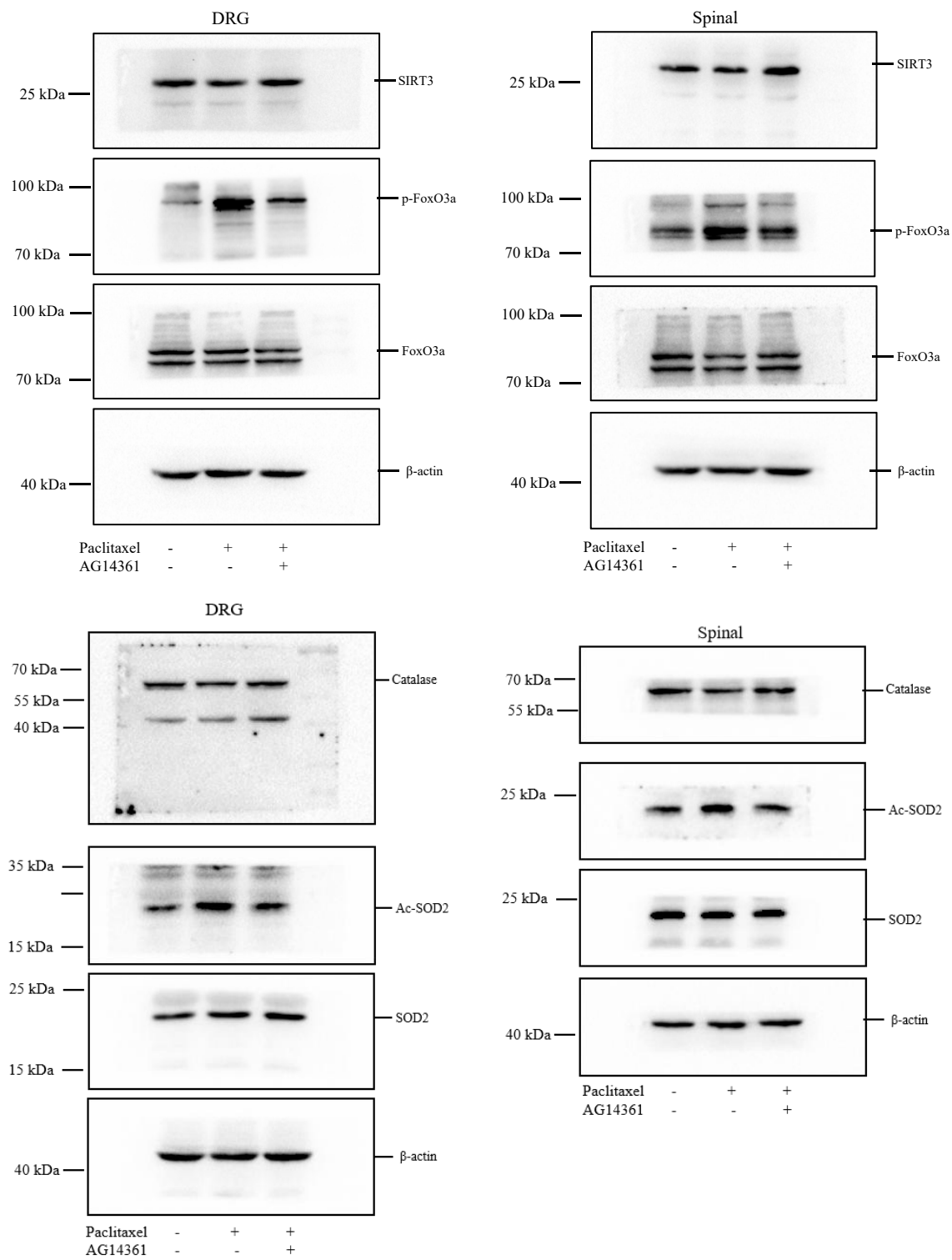

Figure 9

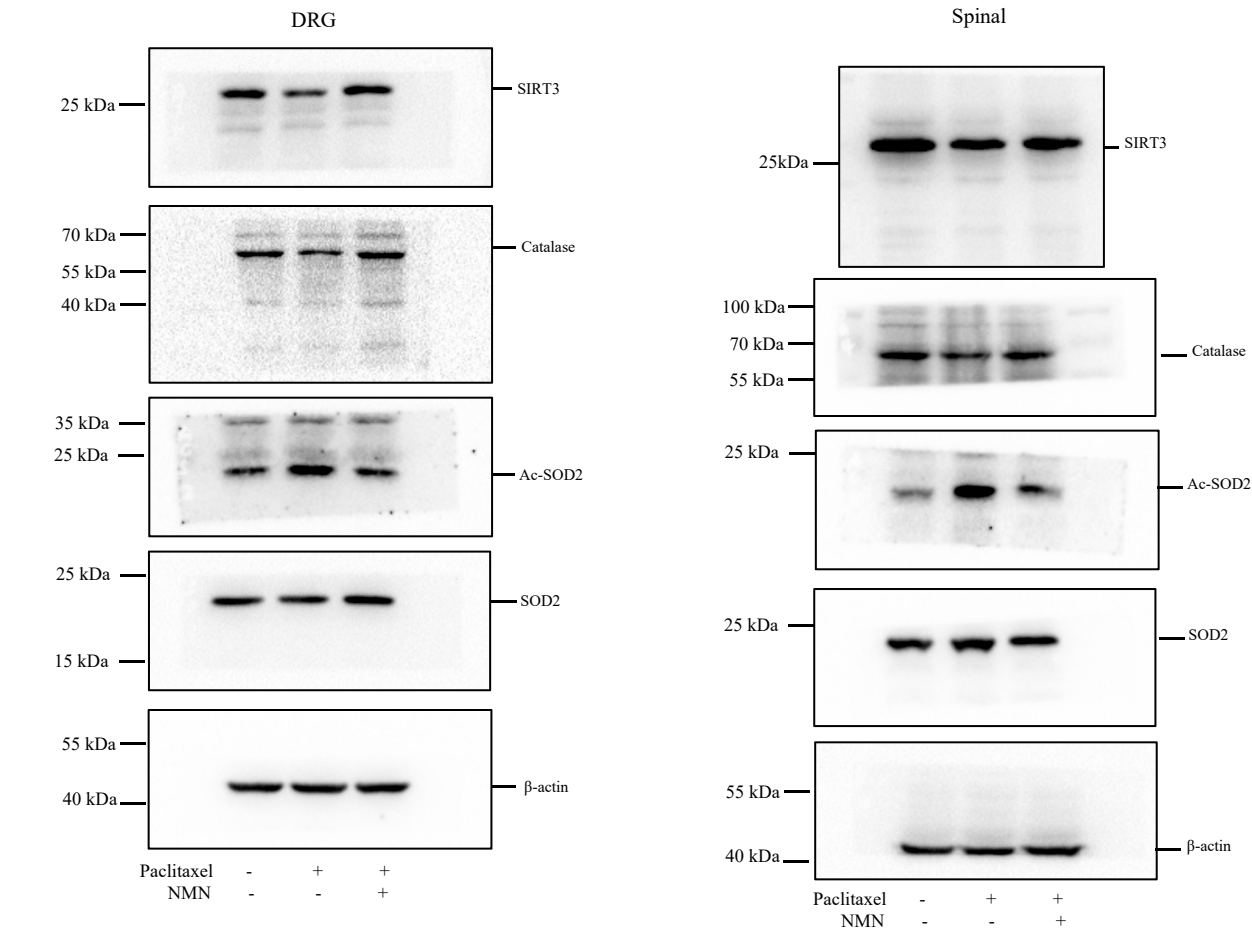

Supplement: Supplementary file 1 — Data S1. [file CNS-30-e70012-s001.zip › Original figures.pdf]
